# Muscidae (Diptera) of forensic importance—an identification key to third instar larvae of the western Palaearctic region and a catalogue of the muscid carrion community

**DOI:** 10.1007/s00414-016-1495-0

**Published:** 2016-12-07

**Authors:** Andrzej Grzywacz, Martin J. R. Hall, Thomas Pape, Krzysztof Szpila

**Affiliations:** 1 Faculty of Biology and Environmental Protection, Nicolaus Copernicus University, Lwowska 1, 87-100 Toruń, Poland; 2 Department of Life Sciences, Natural History Museum, London, UK; 3 Natural History Museum of Denmark, University of Copenhagen, Copenhagen, Denmark

**Keywords:** Forensic entomology, Muscidae, Immature stages, Identification, Post-mortem interval

## Abstract

The Muscidae is one of the main dipteran families recognized as important for medico-legal purposes. Although an association of adult flies with decomposing human and animal bodies is documented for about 200 taxa worldwide, cadavers and carrion represents a breeding habitat for considerably fewer species. Species that do colonize dead human bodies can do so under diverse environmental conditions and, under certain circumstances, Muscidae may be the only colonizers of a body. Because of difficulties in identification, many studies have identified immature and/or adult muscids only to the genus or family level. This lack of detailed species-level identifications hinders detailed investigation of their medico-legal usefulness in carrion succession-oriented experiments. Identification to species level of third instars of Muscidae of forensic importance and the utility of larval morphological characters for taxonomic purposes were subjected to an in-depth revision. A combination of characters allowing for the discrimination of third instar muscids from other forensically important dipterans is proposed. An identification key for third instar larvae, which covers the full set of cadaver-colonising species of Muscidae from the western Palaearctic (Europe, North Africa, Middle East), is provided. This key will facilitate more detailed and species-specific knowledge of the occurrence of Muscidae in forensic entomology experiments and real cases. The carrion-visiting Muscidae worldwide are catalogued, and those species breeding in animal carrion and dead human bodies are briefly discussed with regard to their forensic importance.

**Electronic supplementary material:**

The online version of this article (doi:10.1007/s00414-016-1495-0) contains supplementary material, which is available to authorized users.

## Introduction

Insects often play a major role in the decomposition of organic matter. Generally, the most common arthropod inhabitants of decomposing human cadavers and animal carrion are the larvae of flies (Diptera), particularly those of the families Calliphoridae, Sarcophagidae, Muscidae and Piophilidae [1]. Valuable conclusions for forensic investigations can be drawn from the analysis of entomological material, either by means of age estimation of the oldest immature insects inhabiting the cadaver or by an analysis of arthropod species composition on the body [2].

The Muscidae, commonly known as the house flies and their relatives, is one of the dipteran families of recognized forensic importance. Some textbooks still consider the Fanniidae or lesser house flies as a subfamily within the Muscidae [3–5], but substantial evidence has shown that they warrant family status [6, 7]. Muscids are small- to medium-sized dipterans that can be found in a variety of terrestrial and aquatic habitats, except for the most arid environments [8]. The association between man and Muscidae, for example *Musca domestica* Linnaeus and *M. sorbens* Wiedemann, is traceable to the earliest times of recorded history [9, 10]. Due to their worldwide distribution and broad association with human settlements (many are synanthropic species), muscid flies are renowned for their agricultural, medical and veterinary significance [5, 8]. Most Muscidae hatch from the egg into a first instar larva, which, after feeding for some time, moults into a second and subsequently a third instar before pupariating (so-called trimorphic condition). However, in species of some genera, the incubation period of the egg is prolonged, and the larva hatches from the egg as a second or third instar (dimorphic and monomorphic conditions, respectively). The reduction of free-living larval instars applies only to species with obligatory carnivorous larvae, whereas facultative carnivores are always trimorphic and can reach maturity as non-predators [8].

There are a number of papers in which Muscidae have been discussed in relation to forensic entomology experiments as well as to real cases. Since the pioneer work of Mégnin [11], who reported an association of certain muscids with decomposing bodies, many further muscid taxa have been documented to be attracted to dead human and animal bodies (see Electronic supplementary material 1). Although fewer muscids are known to colonize animal carrion and human cadavers than species of other families, particularly Calliphoridae [12], those that do colonize them can do so under diverse environmental conditions. For example, corpses may be colonized out- or indoors, in sunny or shaded sites, in wet or dry ones, in exposed or concealed situations, and muscids can be found associated with carcasses in both the early and late stages of decomposition.

Muscids have received limited attention in forensic entomology experiments mainly because of taxonomic issues. In some studies, adults and/or immatures were identified to the genus or family level only [13–15]. However, where studies succeeded in identifying species and determining their abundance, both adults and larvae of Muscidae were shown to be very numerous [12, 16, 17]. Species-level identification of entomological material is a prerequisite for a meaningful application of entomological methods for PMI estimation purposes. Thus, when taxonomic complexities or lack of identification tools prevent relatively easy and precise species identification, a broad application of the group for medico-legal purposes is severely limited. The growing sophistication in forensic entomology methodology has raised interest in the larval morphology of dipterans colonising cadavers [18–21]. Although some of these studies concern muscid species as well, they provide no new information and focus solely on the identification of a very few species [19, 21].

The aim of the present study is to provide a key allowing the identification of the third instar larvae of Muscidae breeding in carrion and dead human bodies in the western Palaearctic region (Europe, North Africa and the Middle East). Thus, we catalogue all carrion-visiting Muscidae worldwide and recognize those species or taxa that additionally breed on/in carrion. Subsequently, species breeding in animal carrion and dead human bodies, and therefore of potential forensic importance, were investigated for their geographical distribution, and a detailed morphological study is presented for species of the western Palearctic. Larval morphological characters used by previous authors for taxonomic purposes are subjected to an in-depth revision with the application of the combined methods of light and scanning electron microscopy. We provide a set of characters allowing for the discrimination of larvae of Muscidae from those of other forensically relevant families, and a key is provided for the identification of all studied species. Finally, the role of Muscidae in the faunal succession of cadavers and their application for medico-legal purposes is briefly discussed.

## Material and methods

The selection of species for the present study involved two criteria. First, species visiting carrion and cadavers were identified on the basis of the available literature and communications with practicing forensic entomologists. A taxon was recognized as of potential forensic importance if there was at least one report of immature stages breeding in a human cadaver or in animal carrion. Second, the geographic distribution of each species was studied in the literature data [22–26], and only species confirmed as occurring in the western Palaearctic were included in the study.

Female muscids were collected from the field by hand-netting and the use of carrion-baited traps, and larvae were obtained by keeping those flies in the laboratory until oviposition. Specimens were reared, killed and preserved as described by Grzywacz et al. [27, 28], Grzywacz and Pape [29] and Velásquez et al. [26]. A laboratory colony of *Hydrotaea aenescens* (Wiedemann) was established from adults emerged from *c*. 25 pupae obtained from the Institut de Recherche Criminelle de la Gendarmerie, Fort de Rosny, France. Third instar larvae of *Synthesiomyia nudiseta* (van der Wulp) were obtained from a laboratory colony maintained at the Department of Environmental Sciences and Natural Resources, University of Alicante, Spain, and from the Mexican-American Commission for the Eradication of Screwworm (COMEXA), Chiapa de Corzo, Chiapas, Mexico. Since attempts to obtain immature stages of *Musca autumnalis* De Geer and *Morellia* Robineau-Desvoidy failed, i.e. the collected females either did not oviposit or did not fully develop their eggs, third instar larvae were collected directly from cow manure and identified according to Stoffolano [30] and Skidmore [8]. Larvae of *Musca sorbens* were obtained from the collection of the Natural History Museum, London, UK. The number of examined specimens is as follows: *Atherigona orientalis* Schiner *n =* 16, *Helina* sp. *n =* 36, *Hydrotaea aenescens n =* 476, *H. armipes* (Fallén) *n =* 20, *H. capensis* (Wiedemann) *n =* 45, *H. dentipes* (Fabricius) *n =* 602, *H. ignava* (Harris) *n =* 512, *H. pilipes* Stein *n =* 32, *H. similis* Meade *n =* 20, *Morellia* sp. *n =* 3, *Musca autumnalis n =* 27, *M. domestica n =* 61, *M. sorbens n =* 5, *Muscina levida* (Harris) *n =* 154, *M. prolapsa* (Harris) *n =* 38, *M. stabulans* (Fallén) *n =* 165, *Phaonia* sp. *n =* 41, *Stomoxys calcitrans* (Linnaeus) *n =* 27, and *Synthesiomyia nudiseta n =* 158.

Material for SEM examination was prepared by dehydration through 80.0, 90.0 and 99.5% ethanol, with subsequent critical point drying in CO_2_. Subsequently, larvae were mounted on aluminium stubs and sputter-coated with platinum or gold. SEM images were taken with a JEOL scanning electron microscope (JSM-6335F; JEOL Ltd, Tokyo, Japan) or a variable pressure SEM LEO 1455 (Carl Zeiss Microscopy, Germany).

Light microscopy was performed with a Stemi 2000 stereomicroscope (Carl Zeiss Light Microscopy, Germany). Larvae were mounted on microscope slides in Hoyer’s medium and examined with a Nikon Eclipse E200 microscope (Nikon Corp., Tokyo, Japan). For additional observation of details of the cephaloskeleton, larvae were dehydrated through a 80.0, 96.0 and 99.5% ethanol series and studied with a stereomicroscope 3 h after transfer to methyl salicylate [31]. After examination, the material was transferred back to a 70% ethanol solution.

Photographs for light microscopy illustrations were taken with a Nikon 8400 digital camera mounted either on a Nikon Eclipse E200 microscope or Nikon SMZ 1500 stereomicroscope (Nikon Corp., Tokyo, Japan). Line drawings were prepared by hand and subsequently digitized.

## Results

Searching the available literature revealed 168 muscid species and 31 taxa identified to genus level only that visit dead human bodies or animal carrion worldwide (Electronic supplementary material 1). We expect that the many taxa identified to genus level only are represented among those identified to species level. Decomposing animal carrion and human bodies have been documented as breeding habitats for considerably fewer species, since our search revealed only 25 muscid species and 8 taxa identified to genus level documented from immature stages in forensic case reports and carrion succession experiments. Among these, 14 species and 3 taxa identified to genus level occur in the western Palaearctic region. The present key covers all muscids reported from immature stages from animal carrion and human bodies in the western Palaearctic. Except for *Hydrotaea chalcogaster* (Wiedemann), *H. obscurifrons* (Sabrosky) and *H. spinigera* (Stein), for which we had no material available, the key will work for the entire Holarctic carrion-breeding muscid fauna.

### Identification key to third instar larvae of western Palaearctic Muscidae of forensic importance

**Fig. 1:**
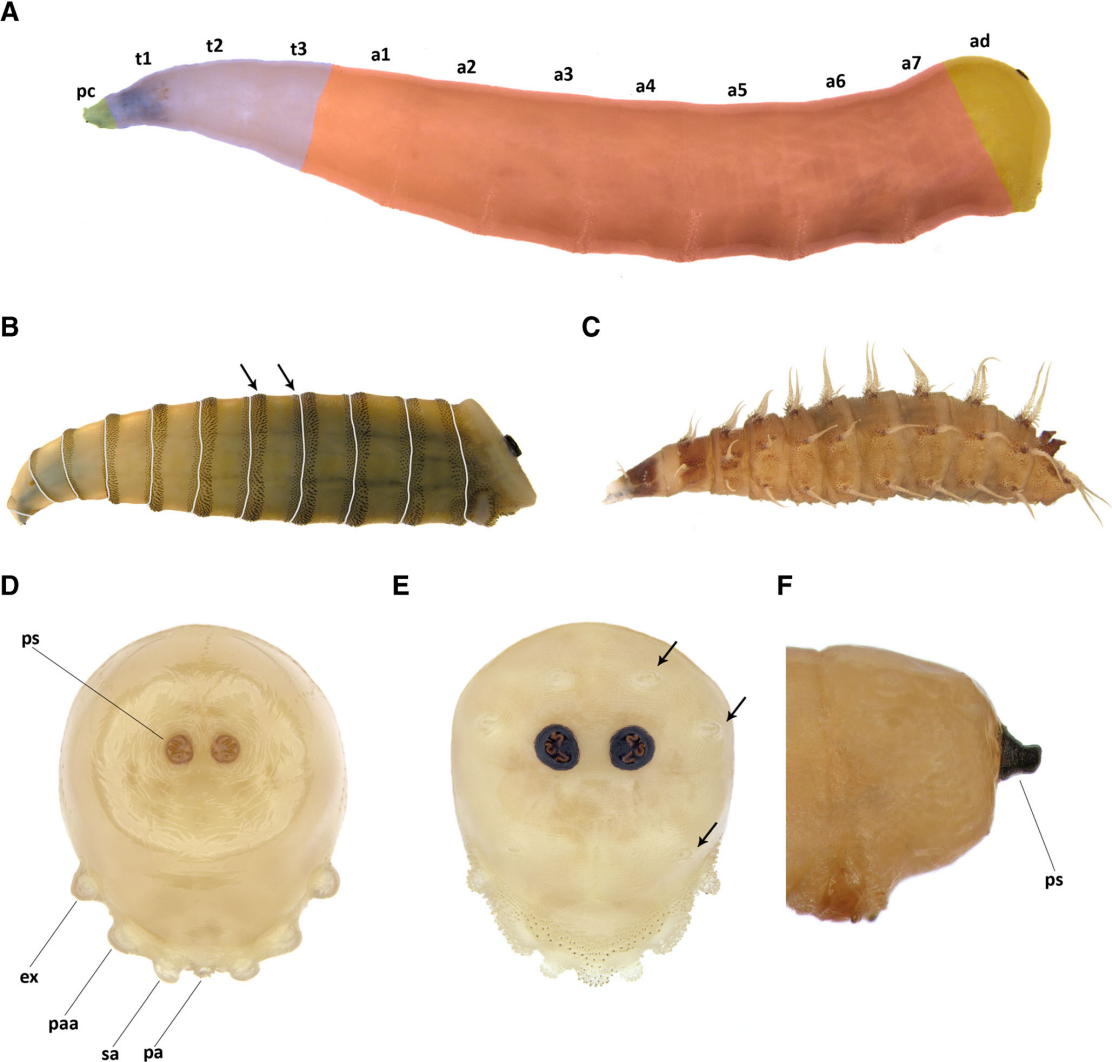
Third instar larvae: **a** *Musca domestica* with artificial colours; **b** *Morellia* sp. with artificial *grey lines* indicating segment borders and *arrows* pointing to the spines on anterior and posterior margins; **c** *Fannia canicularis*; **d** *Hydrotaea dentipes*, anal division in posterior view; **e** *Synthesiomyia nudiseta*, anal division with papillae surrounding posterior spiracles (*arrows*) in posterior view; **f** *Atherigona orientalis*, anal division, lateral view. Abbreviations: *a1–7* abdominal segments, *ad* anal division, *ex* extra-anal papilla, *pa* postanal papilla, *paa* para-anal papilla, *pc* pseudocephalon, *ps* posterior spiracle, *sa* subanal papilla, *t1–3*, thoracic segments

1. Fleshy projections covering the larval body absent and body not flattened dorso-ventrally (Fig. 1a), parastomal bars as well as distinct windows in both dorsal and ventral cornua not developed (Fig. 2a). Unpaired sclerite, either reduced or well developed, present between basal parts of mouthhooks (Fig. 2b–d) → Muscidae 2

**Fig. 2:**
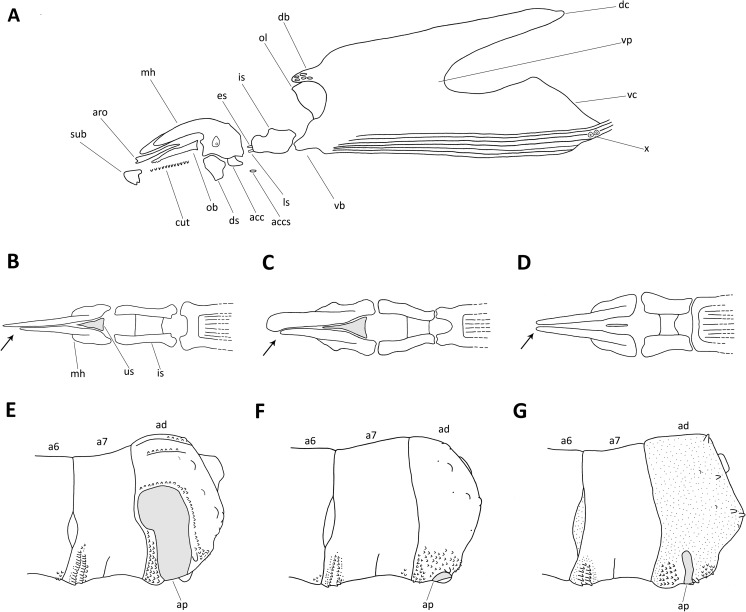
Third instar larvae of Muscidae: **a** structures present in Muscidae cephaloskeleton; **b** cephaloskeleton of predatory species in dorsal view with asymmetric mouthhooks (*arrow*) and well-developed unpaired sclerite (us, *grey*); **c** cephaloskeleton of saprophagous species in dorsal view with asymmetric mouthhooks (*arrow*) and well-developed unpaired sclerite (*grey*); **d** cephaloskeleton of predatory species in dorsal view with symmetric mouthhooks (*arrow*) and reduced unpaired sclerite (*grey*); **e** *Musca autumnalis*, posterior body end with enlarged and broad anal plate (ap, *grey*); **f** *Musca domestica*, posterior body end with small anal plate (ap, *grey*); **g** *Musca sorbens*, posterior body end with enlarged but narrow anal plate (*grey*); Abbreviations: *a6–7* sixth and seventh abdominal segments, *acc* accessory stomal sclerite, *accs* supplementary accessory stomal sclerite, *ad* anal division, *ap* anal plate, *aro* anterior rod, *cut* cutaneous teeth, *db* dorsal bridge, *dc* dorsal cornu, *ds* dental sclerite, *es* epistomal plate, *is* intermediate sclerite, *ls* labial sclerite, *mh* mouthhook, *ob* oral bar, *ol* optic lobe, *sub* suprabuccal teeth, *us* unpaired sclerite, *vc* ventral cornu, *vb* ventral bridge, *vp* vertical plate, *x* sensory organ X
  Fleshy projections covering the larval body present (Fanniidae) (Fig. 1c), if absent then parastomal bars and/or distinct windows in both dorsal and ventral cornua present; mouthhooks always symmetric, accessory stomal sclerites absent, respiratory slits in posterior spiracles never serpentine to tortuous → other forensically important Diptera
2. Posterior spiracles distinctly raised on darkened stalks (Fig. 1f), slits in posterior spiracles peripheral, bow-shaped, never straight, mouthhooks always symmetric (cf. Fig. 2d) → *Atherigona orientalis*
  Posterior spiracles at most slightly raised, if markedly then stalks not darkened and slits straight (Fig. 3a–d), mouthhooks symmetric or asymmetric (Fig. 2b–d) → 3

**Fig. 3:**
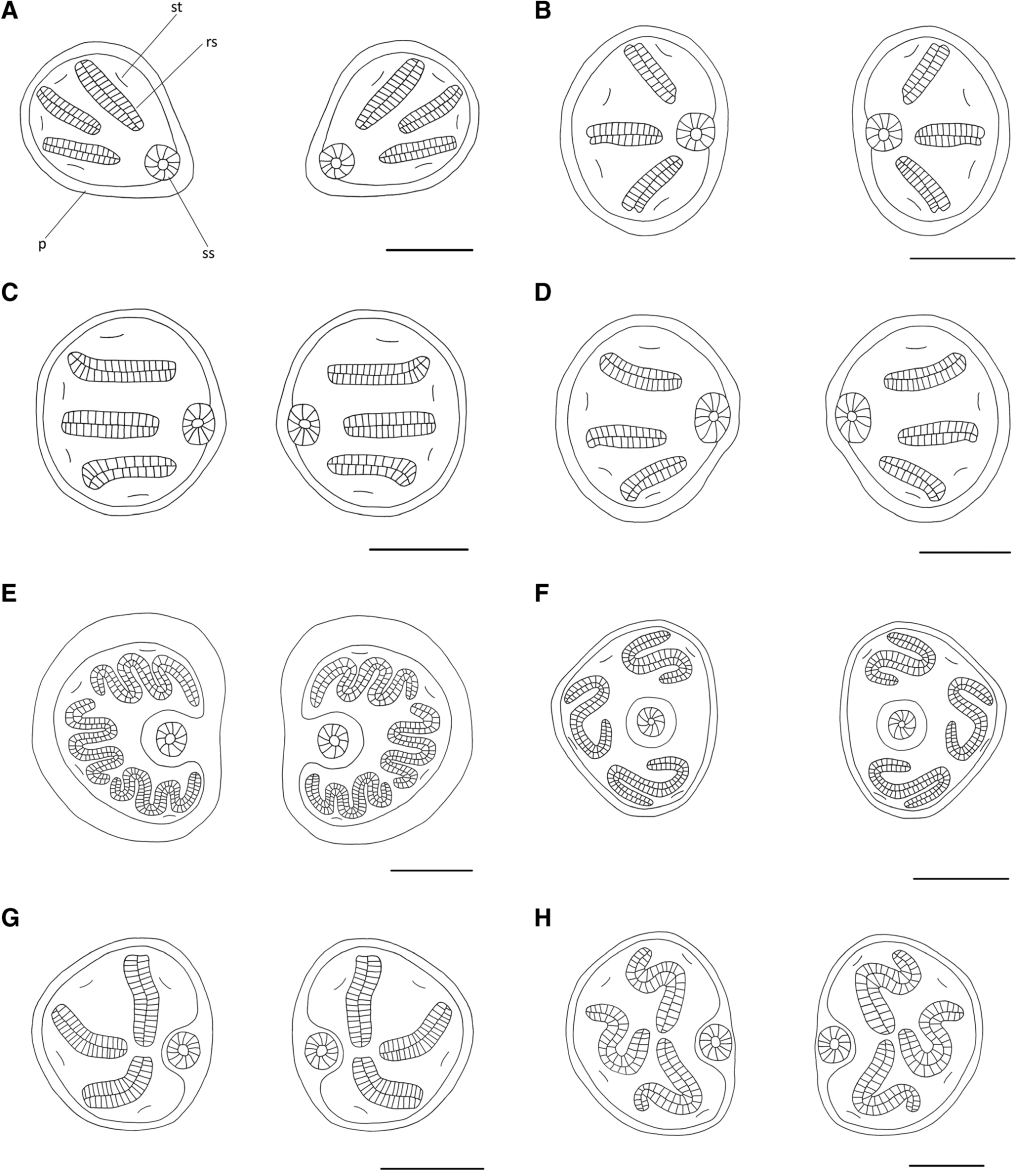
Posterior spiracles of Muscidae third instar larvae of forensic interest: **a** *Hydrotaea dentipes*; **b** *H. pilipes*; **c** *H. capensis*; **d** *H. ignava*; **e** *Musca domestica*; **f** *Stomoxys calcitrans*; **g** *Muscina stabulans*; **h** *Synthesiomyia nudiseta. Scale bar* 0.1 mm. Abbreviations: *p* peritreme, *rs* respiratory slit, *ss* spiracular scar, *st* spiracular tuft
3. Anal division abruptly truncated, abdominal segments 1–7 covered by complete anterior and posterior bands of dark spines (Fig. 1b) → *Morellia* sp.
  Abdominal segments 1–7 without complete posterior bands of spines → 4
4. Slits in posterior spiracles serpentine (Fig. 3f–h) to tortuous (Fig. 3e); peritreme surrounding respiratory slits often darkly pigmented (Fig. 1e) → 5
  Slits of posterior spiracles straight, never serpentine (Fig. 3a–d); peritreme often clear and poorly pigmented (Fig. 1d) → 12
5. Posterior spiracles subtriangular (i.e. almost triangular in shape, but with rounded corners), respiratory slits S-shaped encircling spiracular scar and the scar shifted towards central position (Fig. 3f) → *Stomoxys calcitrans*
  Posterior spiracles not subtriangular, kidney-shaped (Fig. 3e) or rounded (Fig. 3e, g, h), respiratory slits not encircling spiracular scar and the scar placed in median position (Fig. 3e, g, h) → 6
6. Posterior spiracles kidney-shaped (Fig. 3e); extra-anal and postanal papillae on anal division absent and subanal papillae devoid of spines (for papillae position see Fig. 1d); anterior rods and oral bars absent, suprabuccal teeth devoid of distinct pigmentation, optic lobe present (Fig. 2a), dental sclerites separated ventrally, mouthhooks asymmetric (Fig. 2c) → *Musca* sp. 7
  Posterior spiracles rounded (Fig. 3g, h); extra-anal and postanal papillae on anal division well developed and subanal papillae covered by spines (Fig. 1e); mouthhooks symmetric (Fig. 2d), anterior rods and oral bars well developed, suprabuccal teeth distinctly dark pigmented, optic lobe absent (Fig. 2a), dental sclerites joined ventrally → 9
7. Anal plate small, barely visible in lateral view (Fig. 2f); dorsal surface of the anal division devoid of spines and papillae surrounding posterior spiracles indistinguishable (Fig. 1a) → *Musca domestica*
  Anal plate well visible in lateral view (Fig. 2e, g) and papillae surrounding posterior spiracles well visible, bulge or cone-shaped → 8
8. Anal plate large, well visible in lateral view (Fig. 2e); dorsal surface of the anal division covered by spines → *Musca autumnalis*
  Anal plate narrow, not angular (Fig. 2g); anal division covered entirely with minute spicules → *Musca sorbens*
9. Slits in posterior spiracles S-shaped (Fig. 3h); anal plate small, triangular in ventral view; para-anal papillae well developed; papillae surrounding posterior spiracles cone-shaped (Fig. 1e) → *Synthesiomyia nudiseta*
  Slits crescent shaped (Fig. 3g); anal plate extended laterally in ventral view; para-anal papillae indistinguishable; papillae surrounding posterior spiracles at most in the form of small bulges (cf. Fig. 1d) → *Muscina* spp. (for details see Grzywacz et al. [28]) 10
10. Postero-ventral surface of abdominal segments 5–6 at most with a single up to two rows of about five spines each (Figs. 1f, 7B in Grzywacz et al. [28]) → *Muscina prolapsa*
  Postero-ventral surface of abdominal segments 5–6 with about ten rows of about five small, light spines each (Figs. 1e, g and 4d; 9B in Grzywacz et al. [28]) → 11

**Fig. 4:**
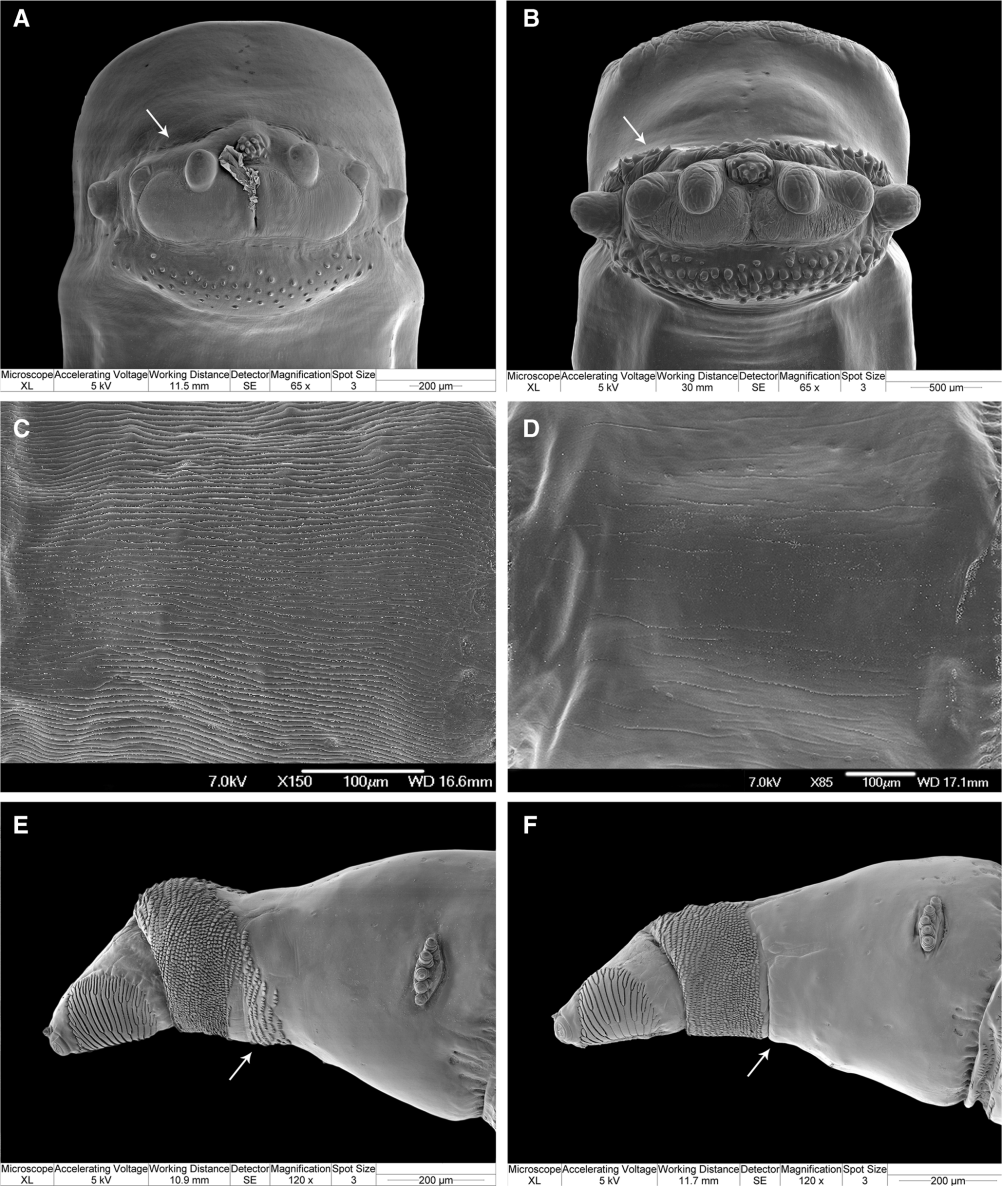
Scanning electron microscopy images of the third instar larval morphology of Muscidae of forensic interest: **a** *Hydrotaea dentipes*, posterior body end in ventral view with posterior surface, behind anal papillae, devoid of spines (*arrow*); **b** *H. similis*, posterior body end in ventral view with posterior surface, behind anal papillae, covered with spines (*arrow*); **c** *H. armipes*, body surface of the first abdominal segment; **d** *H. pilipes*, body surface of the first abdominal segment; **e** *H. aenescens*, anterior body end, lateral view with spinose band on the first thoracic segment not uniformly broad (*arrow*); **f** *Hydrotaea capensis*, anterior body end, lateral view with the spinose band on the first thoracic segment uniformly broad (*arrow*)
11. Spines on the lateral surface of the third abdominal segment reaches well above the upper margin of the lateral creeping welt (Fig. 1e, 4a in Grzywacz et al. [28]) and anterior spinose band on the fourth abdominal segment (a4) reaches at least the middle of lateral creeping welt → ***Muscina levida***
  Spines on the lateral surface of the third abdominal segment reaches at most slightly above the lateral cree-ping welt (Fig. 1g, 9A in Grzywacz et al. [28]) and anterior spinose band on the fourth abdominal segment hardly present (Fig. 1g in Grzywacz et al. [28]) → *Muscina stabulans*
12. Mouthhooks symmetric (Fig. 2d) → *Helina* spp. & *Phaonia* spp.
  Mouthhooks asymmetric (Fig. 2b) → *Hydrotaea* sp. 13
13. Posterior spiracles ovoid, spiracular scar in inferior position (Fig. 3a) → 14
  Posterior spiracles rounded, scar in middle position (Fig. 3b–d) → 15
14. Posterior surface of anal division, behind anal papillae, devoid of spines (Fig. 4a, arrow) → *Hydrotaea dentipes*
  Posterior surface of anal division, behind anal papillae, covered by spines (Fig. 4b, arrow) → *Hydrotaea similis*
15. Surface of the anal division surrounding anal papillae smooth, without distinct spines; complete spinose band present only on the first thoracic segment; virtual projections of respiratory slits in posterior spiracles distinctly convergent (Fig. 3b) → 16
  Surface of the anal division surrounding anal papillae covered with distinct spines; complete spinose bands not restricted to the first thoracic segment; slits of posterior spiracles parallel (Fig. 3c) or slightly convergent (Fig. 3d) → 17
16. Larval body covered with distinct longitudinal ridges (Fig. 4c) → *Hydrotaea armipes*
  Larval body smooth, devoid of distinct longitudinal ridges (Fig. 4d) → *Hydrotaea pilipes*
17. Spinose band on the first thoracic segment not uniformly broad, broadened ventrally by an additional patch of spines (Fig. 4e, arrow) → *Hydrotaea aenescens*
  Spinose band on the first thoracic segment uniformly broad (Fig. 4f, arrow) → 18
18. Upper and lower respiratory slits in posterior spiracles parallel (Fig. 3c), at most slightly convergent towards the median scar → *Hydrotaea capensis*
  Upper and lower respiratory slits in posterior spiracle distinctly convergent towards the median scar (Fig. 3d) → *Hydrotaea ignava*

## Discussion

### Forensically important species

Muscid flies have been reported in numerous forensic studies that either describe the succession of insects on carrion or are inventories of local carrion faunas. Due to problems with identification, in many of these studies, Muscidae are referred to at the genus or family level only [13, 14, 32–37], which may give the impression of low diversity. However, when authors have attempted to identify muscids to species, it often emerged that they were very numerous and diverse [12, 17, 38–40].

The present study demonstrated that more than 150 species of Muscidae have been reported to visit either human bodies or animal carrion (Electronic supplementary material 1). We were cautious with our pooling of literature data to avoid an overestimation of the number of carrion-visiting Muscidae taxa. For example, Alves et al. [41] miscalculated carrion-visiting muscids in South America, erroneously listing *Hydrotaea aenescens* and its two junior synonyms *H. argentina* (Bigot) and *Ophyra argentina* as well as *Synthesiomyia nudiseta* and its junior synonym *S. brasiliana* Brauer & Bergenstamm as three and two valid species, respectively, rather than as just two species overall. For the majority of species reported to be visiting carrion as adults, carrion is not documented as a breeding medium. Hence, immature stages of only 25 muscid species and 8 taxa identified to genus level only have been found to develop in this habitat, either feeding on the putrefying tissues or preying on other necrophagous larvae. Some Muscidae that are predatory in the larval stage reside in the soil beneath or close to cadavers (e.g. *Phaonia* Robineau-Desvoidy, *Helina* Robineau-Desvoidy) and may erroneously be considered as a component of the carrion fauna, when they are actually preying on larvae dispersing from the cadaver. The most regular and frequent muscid components of the carrion-breeding community are species of the genera *Atherigona* Rondani, *Hydrotaea* Robineau-Desvoidy, *Musca* Linnaeus, *Muscina* Robineau-Desvoidy and *Synthesiomyia* Brauer & Bergenstamm. Some of these species have a wide geographic distribution and have been reported as elements of carrion communities in different regions of the world, e.g. *A. orientalis*, *H. aenescens*, *H. capensis*, *H. ignava*, *H. dentipes*, *M. domestica*, *M. stabulans*.

In the family Muscidae, some species have recently been shown to be common components of the carrion fauna [16], while many others are just casual visitors as adults [12, 16], attracted less frequently/predictably to decomposing tissues for feeding purposes. Furthermore, Matuszewski et al. [16] revealed that even regular carrion-visiting species may be of little or no forensic value, and the medico-legal usefulness of each taxon requires a detailed study before a firm assessment can be made of its potential forensic significance. However, researchers should be aware of the possible occurrence among the typical carrion-visiting and carrion-breeding species of numerous more rarely attracted taxa. This may be the case for some of the Muscidae listed here, since for about 80 taxa, we found only a single reference reporting the presence of adults on carrion (Electronic supplementary material 1). For this reason, application of identification keys for adult muscids with a broad taxon coverage for the geographic region of interest is recommended in forensic entomology surveys instead of those exclusively oriented towards identification of ‘forensically important’ species [42].

We included all references to carrion-visiting muscids that we are aware of, but may have missed some less obvious reports; nevertheless, we consider the set of western Palaearctic taxa presented here, particularly the narrow set of carrion breeders, to be complete. However, we expect that further species will be named that are either rare visitors as adults or breed in carrion and cadavers, in particular in the latter group from the genera *Azelia* Robineau-Desvoidy and *Morellia*.

### Muscidae in forensic context

The larvae of muscids that are commonly considered as forensically important are either truly necrophagous or display a predacious behaviour as they mature. In the latter case (e.g. *A. orientalis*, *Hydrotaea* spp., *Muscina* spp., *S. nudiseta*), they can considerably lower the abundance of other necrophagous species by preying on their larvae, similar to some predatory blow flies and flesh flies [1]. Muscidae are considered to arrive at cadavers and carrion just after the blow flies and flesh flies [5]. However, in some cases, muscids were the only insects reported to colonize decomposing bodies, especially if access to a corpse was denied to the primary carrion colonizers [1]. Muscids are generally considered to breed in carrion in the later stages of decomposition, and they tend to occur at the moist advanced decay stage [5]. Under certain circumstances, muscids can occur on cadavers as pioneer colonizers. Smith [1] stated that *Musca autumnalis* usually occurs in the early stages of cadaver decomposition and reported a similar occurrence for representatives of the genus *Muscina*, but according to Thomson [43], species of the latter genus prefer cadavers already colonized by other flies. Certain synanthropic species, e.g. *Musca domestica* and *Muscina stabulans*, are likely to be associated with cadavers in domestic conditions and under certain circumstances may be the sole colonizers of a body [1]. The majority of species are not synanthropic and probably do not inhabit human dwellings, being associated instead with rural and forest habitats [12, 16]. Muscidae are not frequently referred to in forensic studies, despite the fact that many Muscidae are regularly attracted to carrion (Electronic supplementary material 1). However, recently, some authors have revealed a high diversity of Muscidae among arthropods attracted to decomposing carrion in rural and forest habitats of Central Europe [12, 16, 44]. In these habitats, muscid species significantly outnumbered Sarcophagidae, commonly considered as one of the most forensically important groups of insects [16, 45]. Matuszewski et al. [16] found a significant association of adults of *H. aenescens*, *H. armipes*, *H. cyrtoneurina* (Zetterstedt), *H. dentipes*, *H. ignava*, *H. pilipes* and *H. similis* and larvae of *H. ignava* and *H. dentipes* with the bloated stage of carrion decomposition.

According to Smith [1], *Musca domestica* and *Muscina* spp. are more readily attracted to bodies contaminated with faeces rather than to those not so contaminated. Indeed, Benecke and Lessig [46] reported child neglect preceding death due to the presence of *M. stabulans* larvae attracted to faeces. The occurrence of Muscidae on a cadaver prior to death is of importance and could happen, not only because the flies were attracted by faeces present on the body but also from infected wounds, because some muscids are known to be involved in cases of secondary myiasis in humans and animals [47], e.g. *M. domestica*, *Muscina levida*, *M. prolapsa*, *M. stabulans* and *S. nudiseta*.

Restricted access of arthropods to a dead body has been recognized as one of the most important factors affecting the breakdown of cadaver. Concealed remains, e.g. buried bodies, can still be colonized by insects, but even a relatively thin layer of soil, just 5–10 cm, may either disturb or inhibit colonization by some typical necrophagous species [48]. Although some authors have reported flies (*Calliphora vicina* Robineau-Desvoidy) ovipositing on the soil covering a body buried at a depth of 30 cm [14], such observations are not consistent with the ability of larvae to reach a buried corpse [48]. Some Muscidae, particularly of the genera *Muscina* and *Hydrotaea*, together with some Phoridae and Sarcophagidae, are among the few dipterans known for their ability to exploit buried remains [20, 37, 48, 49], and in some cases, Muscidae have even been described as predominant on buried remains [49, 50]. Nuorteva [51] observed females of *H. dentipes* ovipositing on a human corpse partly covered with snow, and Anderson [52] reported a *Hydrotaea* sp. colonising a body placed in a car trunk and Shin et al. [53] reported *H. obscurifrons* also from a body in a car trunk. According to Mariani et al. [54], *H. aenescens* and *M. stabulans* are able to develop through several generations on a buried cadaver. A similar phenomenon has been observed for *H. capensis* colonising bodies in buried coffins [55]. Skidmore [8] reported that *H. dentipes* and *H. ignava* overwinter in the larval or pupal stage, and recently, Mądra et al. [44] revealed those two species and *H. pilipes* overwintering on pig carcasses in their immature stages. Two species of Muscidae (*H. capensis*, *M. prolapsa*) have been reported to develop in pig heads concealed in zipped suitcases [56].

### Identification of third instar larvae

Precise species identification of larvae inhabiting dead bodies is a crucial first step in the analysis of insect evidence in any forensic case [2]. The literature concerning larval morphology of Muscidae is extensive, but has a strong bias towards species of sanitary, medical, veterinary and agricultural importance. This bias causes difficulties in the identification of third instar larvae of a broader range of Muscidae, ultimately severely restricting the analysis of such entomological material in forensic cases and carrion succession experiments. Until now, the only reliable alternative method for identification of larval material has been rearing to adults in the laboratory. Although rearing can be simple, it requires carefully sampled live specimens. Also, it takes from 2 to 5 weeks and may be unsuccessful if, for example, the larvae have been contaminated by insecticides or injured through desiccation [51]. Species identification of preserved larvae is therefore of considerable practical importance. Some authors claim that because identification based on morphological methods requires specialized taxonomic knowledge, only some specialists are able to identify larvae of forensically relevant insects to species level [2]. For this reason, other methods of species identification have been developed, such as molecular approaches. However, molecular libraries for identification of Muscidae have not yet been sufficiently developed and currently do not allow identification of the full set of taxa here recognized as breeding in carrion and human cadavers [57–61]. Hence, identification keys based on morphological characters of larvae remain an important tool in forensic entomology. In particular, well-illustrated keys should be developed that are readily available (open access) to the non-entomologist, relying as much as possible on easily recognizable characters.

Species of Muscidae occurring in the western Palaearctic region breeding in carrion and cadavers have been shown here to differ sufficiently in their third instar larval morphology to allow for their discrimination. *Musca sorbens*, known as the bazaar fly, is a species closely associated with humans in all areas of its occurrence. There is no confirmed report of its larvae breeding either in animal carrion or human cadavers. However, the species is closely associated with animal dung and human faeces, and therefore, the presence of its larvae on cadavers contaminated with faeces is possible and the species has been included in the present key. Similar to *M. sorbens* is the case of *Stomoxys calcitrans*. Although this species has not been reported breeding in carrion and cadavers, we found a report of unidentified representative of the genus *Stomoxys* from a human cadaver in France [62]. Because *S. calcitrans* is the only Palaearctic species from this genus and, similarly to *M. sorbens*, is closely associated with human dwellings, we included it in the key. As the genus *Morellia* has only been recorded once from decomposing carrion [14], and then only from North America and without a specific identification, this taxon has been excluded from detailed study. However, because *Morellia* species occurring in the western Palaearctic region share a similar immature biology with the North American fauna, and furthermore one North American representative, *M. podagrica* (Loew), is known from the western Palaearctic, the genus has been included in the identification key provided here. Although larvae of some species of *Helina* and *Phaonia* have been reported from decomposing carrion, they have here been considered as rare visitors without forensic importance. Representatives of *Helina* and *Phaonia* are obligatory predators [8], living e.g. in humus-rich soil or under tree bark. In these habitats, they are active predators that prey on other arthropod larvae. However, due to possible accidental occurrence in and around decomposing cadavers, they have also been included in the identification key.

Some serious discrepancies with the present study and misinterpretations have been revealed in the medical and veterinary entomology literature, including textbooks concerning the identification of third instar larvae of forensically important Muscidae. All these cases are discussed in detail in the attached appendix (Electronic supplementary material 2).

We incorporated in the identification key larval morphology characters widely used hitherto and also others not previously recognized as valuable for taxonomic purposes. Since the presence or absence of spines covering thoracic and abdominal segments in Muscidae may be difficult to observe because of their lack of colour, previous authors did not describe details of the spinulation pattern nor did they include it in identification keys, with few exceptions [63, 64]. Although often difficult to observe and with some intraspecific variability, the spinulation pattern has been recognized as useful for taxonomic purposes, particularly for discrimination of representatives of *Muscina* [28]. Some students of Muscidae third instar morphology identified the importance of the distance separating posterior spiracles for taxonomic purposes [64, 65]. However, this has been revealed insufficient for taxonomic purposes in third instar larvae [28]. On the other hand, valuable characters for identification of third instars of muscids are found in details of posterior spiracles, since the spiracular scar and respiratory slits exhibit great variation in their shape and arrangement (Fig. 3a–h). However, the pigmentation of the posterior spiracles and, in some species, of the adjoining area increases during larval growth, and so it is not a useful character (for details see Grzywacz et al. [28]). The cephaloskeleton, a structure reflecting the larval feeding strategy, differs between saprophagous, and both facultative and obligatory carnivores and is of primary importance for taxonomic purposes (Fig. 2a–d). In species with asymmetric mouthhooks, the apical parts appose closely, appearing as one structure, whereas in those with symmetric mouthhooks, the apical parts adjoin but are clearly separated. The basal parts of mouthhooks in the former group are joined through the broadened unpaired sclerite, while in the latter group, the basal parts are distinctly separated.

## Conclusions

A high species diversity has been revealed for the community of carrion-visiting Muscidae. Recent studies have significantly added to this set of muscids known to be attracted to decomposing carrion/cadavers. Some species have been shown to regularly either visit carrion as adults or to breed in/on decomposing bodies and carrion. Although the value of some muscid species as forensic indicators has been documented, particularly in Central European habitats, the potential value of many other species still needs to be studied in detail. The key for the identification of third instar larvae provided here will allow for precise identification of all muscid species known to breed in human cadavers in the western Palaearctic, facilitating a better understanding of the role played by Muscidae as forensic entomology indicators. Data to estimate the age of the oldest cadaver colonizers, thereby the minimum PMI, includes developmental models of a particular species under specific conditions gathered by laboratory studies. Though such data have been provided for some muscids [26, 66–69], the majority of species still need to be studied.

## Electronic supplementary materials

Below is the link to the electronic supplementary material.

ESM 1 (PDF 100 kb)

ESM 2 (PDF 31.8 kb)
