# Supplementary material for: Muscidae (Diptera) of forensic importance—an identification key to third instar larvae of the western Palaearctic region and a catalogue of the muscid carrion community
Source: Int J Legal Med. 2016 Dec 7;131(3):855–66. doi: 10.1007/s00414-016-1495-0 (PMC5388714; doi:10.1007/s00414-016-1495-0)
Supplement: Supplementary file 1 — (PDF 100 kb) [file 414_2016_1495_MOESM1_ESM.pdf]

Table 1. References for Muscidae visiting and/or breeding in human bodies and/or animal carrion (bolded species and taxa reported in immature stages from carrion and cadavers occurring in the western Palaearctic).

No	Taxon	Author	Distribution	Forensic importance	References
1.	*Alluaudinella flavicornis*	(Macquart, 1855)	AF	AC (A, I)	[1]
2.	*Atherigona longipalpis*	Malloch, 1923	Guam	HC (A)	[2]
3.	***Atherigona orientalis***	Schiner, 1868	AF, A/O, NA, NT, OR, PA	HC (A, I); AC (A, I)	[2–14]
4.	*Atherigona savia*	Pont et Magpayo, 1995	OR	AC (A)	[6]
5.	*Atherigona* sp.	Rondani	AF, A/O, NA, NT, OR, PA	AC (A)	[6, 15–18]
6.	*Australophyra rostrata*	(Robineau-Desvoidy, 1830)	A/O	AC (A, I)	[19–23]
7.	*Azelia aterrima*	(Meigen, 1826)	PA	AC (A)	Matuszewski unpubl.
8.	*Azelia cilipes*	(Haliday, 1838)	NA, PA	AC (A)	[24]
9.	*Azelia nebulosa*	Robineau-Desvoidy, 1830	PA	AC (A)	Jarmusz unpubl.
10.	*Azelia* sp.	Robineau-Desvoidy, 1830	AF, NA, NT, PA, OR	AC (A, I)	[25–27]
11.	*Azelia trigonica*	Hennig, 1956	PA	AC (A)	[24]
12.	*Azelia triquetra*	(Wiedemann, 1817)	NA, PA	AC (A)	[24]
13.	*Azelia zetterstedtii*	Rondani, 1866	NA, PA	AC (A)	[24, 28]
14.	*Biopyrellia bipuncta*	(Wiedemann, 1830)	NT	AC (A, I)	[7, 8, 12, 14, 29, 30]
15.	*Biopyrellia* sp.	Townsend, 1932	NT	AC (A)	[10]
16.	*Bithoracochaeta calopus*	(Bigot, 1885)	NT	AC (A)	[31]
17.	*Bithoracochaeta* sp.	Stein, 1911	NT	AC (A)	[10, 29]
18.	*Brontaea delecta*	(van der Wulp, 1896)	NA, NT, Hawaii	AC (A)	[14, 32]
19.	*Brontaea debilis*	(Williston, 1896)	NA, NT	AC (A)	[7, 10, 14]
20.	*Brontaea normata*	(Bigot, 1885)	NT	AC (A)	[12, 14, 29]
21.	*Brontaea polystigma*	(Meigen, 1826)	PA	AC (A)	[33]
22.	*Brontaea* sp.	Kowarz, 1873	AF, A/O, NA, NT, OR, PA	AC (A)	[7, 12, 14, 27, 34]
23.	*Coenosia agromyzina*	(Fallén, 1825)	PA	AC (A)	Matuszewski unpubl.
24.	*Coenosia attenuata*	Stein In Becker, 1903	A/O, NT, PA, OR	AC (A)	Farinha unpubl.
25.	*Coenosia humilis*	Meigen, 1826	AF, NA, OR, PA	AC (A)	[24]
26.	*Coenosia nigridigita*	Rondani, 1866	PA	AC (A)	[24]
27.	*Coenosia pumila*	(Fallén, 1825)	NA, PT	AC (A)	Matuszewski unpubl.

28.	*Coenosia testacea*	(Robineau-Desvoidy, 1830)	PA	AC (A)	[24]
29.	*Coenosia tigrina*	(Fabricius, 1775)	NA, PT	AC (A)	[24] Matuszewski unpubl.
30.	*Coenosia verralli*	Collin, 1953	NA, PT	AC (A)	Matuszewski unpubl.
31.	*Correntosia bicolor*	Malloch, 1934	NT	AC (A)	[14]
32.	*Cyrtoneurina crispaseta*	Snyder, 1954	NT	AC (A)	[14]
33.	*Cyrtoneurina* sp.	Giglio-Tos, 1893	NT	AC (A, I)	[7, 12, 14, 30, 31]
34.	*Cyrtoneuropsis conspersa*	(Stein, 1911)	NT	AC (I)	[7, 12, 30]
35.	*Cyrtoneuropsis dubia*	(Snyder, 1954)	NT	AC (A)	[14]
36.	*Cyrtoneuropsis gluta*	(Giglio-Tos, 1893)	NT	AC (A)	[14]
37.	*Cyrtoneuropsis incognita*	(Snyder, 1954)	NT	AC (A)	[14]
38.	*Cyrtoneuropsis maculipennis*	(Macquart, 1843)	NT	AC (A)	[14, 31]
39.	*Cyrtoneuropsis pararescita*	(Couri, 1995)	NT	AC (A)	[14]
40.	*Cyrtoneuropsis rescita*	(Walker, 1861)	NT	AC (A)	[7, 35]
41.	*Cyrtoneuropsis similata*	(Couri, 1982)	NT	AC (A)	[14]
42.	*Cyrtoneuropsis* sp.	Malloch, 1925	NT	AC (A)	[10]
43.	*Cyrtoneuropsis varicolor*	(Hough, 1900)	NT	AC (A)	[14]
44.	*Cyrtoneuropsis veniseta*	(Stein, 1904)	NT	AC (A)	[14]
45.	*Dasyphora albofasciata*	(Macquart, 1839)	PA	AC (A)	[27]
46.	*Dasyphora pratorum*	(Meigen, 1826)	PA	AC (A)	[33]
47.	*Dichaetomyia saperoi*	Bohart et Gressitt, 1946	A/O	HC (A)	[2]
48.	*Dichaetomyia nigroscuta*	Bohart et Gressitt, 1946	A/O	HC (A)	[2]
49.	*Dichaetomyia* sp.	Malloch, 1921	A/O, PA, OR	HC (A)	[16]
50.	*Eudasyphora cyanella*	(Meigen, 1826)	AF, PA	AC (A)	[36–38]
51.	*Eudasyphora cyanicolor*	(Zetterstedt, 1845)	NA, PA	AC (A)	[28, 33, 39] Jarmusz unpubl.
52.	*Eudasyphora* sp.	Townsend, 1911	NA, PA	AC (A)	[27]
53.	*Graphomya analis*	(Macquart, 1851)	NA	AC (A)	[14]
54.	*Graphomya maculata*	(Scopoli, 1763)	A/O, OR, PA	AC (A)	[7, 12, 24, 37]
55.	*Graphomya mexicana*	Giglio-Tos, 1893	NT	AC (A)	[10]
56.	*Graphomya minor*	Robineau-Desvoidy, 1830	PA	AC (A)	Jarmusz unpubl., Matuszewski unpubl.
57.	*Graphomya* sp.	Robineau-Desvoidy, 1830	AF, A/O, NA, NT, OR, PA	AC (A)	[8, 27, 40]
58.	*Haematobia irritans*	(Linnaeus, 1758)	Hawaii, NA, NT, OR, PA	AC (A)	[29]

59.	*Haematobosca stimulans*	(Meigen, 1824)	OR, PA	AC (A)	Matuszewski unpubl.
60.	*Hebecnema fumosa*	(Meigen, 1826)	OR, PA	AC (A)	[41]
61.	*Hebecnema nigra*	(Robineau-Desvoidy, 1830)	NA, PA	AC (A)	[24] Matuszewski unpubl.
62.	*Hebecnema* sp.	Schnabl, 1889	A/O, NA, PA	AC (A)	[27]
63.	*Hebecnema umbratica*	(Meigen, 1826)	NA, OR, PA	AC (A)	[24]
64.	*Hebecnema vespertina*	(Fallén, 1823)	NA, PA	AC (A)	[24]
65.	*Helina allotalla*	(Meigen, 1830)	PA	AC (A)	[42]
66.	*Helina annosa*	(Zetterstedt, [1838])	NA, PA	AC (A)	Jarmusz unpubl.
67.	*Helina deleta*	(Stein, 1914)	PA	AC (A)	Jarmusz unpubl.
68.	*Helina depuncta*	(Fallén, 1825)	PA	AC (A)	[37, 38]
69.	*Helina evecta*	(Harris, [1780])	AF, NA, NT, OR, PA	AC (A)	[39, 41] Matuszewski unpubl.
70.	*Helina impuncta*	(Fallén, 1825)	PA	AC (A, I)	[24, 36–38]
71.	*Helina latitarsis*	Ringdahl 1924	PA	AC (A)	[24]
72.	*Helina laxifrons*	(Zetterstedt, 1860)	NA, PA	AC (A)	Matuszewski unpubl.
73.	*Helina obscurata*	(Meigen, 1826)	NA, PA	AC (A)	[39]
74.	*Helina parcepilosa*	(Stein, 1907)	PA	AC (A)	Matuszewski unpubl.
75.	*Helina pubescens*	(Stein, 1893)	PA	AC (A)	[38] Matuszewski unpubl.
76.	*Helina quadrum*	(Fabricius, 1805)	PA	AC (A)	Matuszewski unpubl.
77.	*Helina reversio*	(Harris, [1780])	NA, PT	AC (A)	[24, 38, 39] Matuszewski unpubl.
78.	*Helina sexmaculata*	(Preyssler, 1791)	A/O, NA, OR, PA	AC (A)	[24]
79.	*Helina trivittata*	(Zetterstedt, 1860)	PA	AC (A)	Matuszewski unpubl.
80.	***Helina* sp.**	Robineau-Desvoidy, 1830	AF, A/O, NA, NT, OR, PA	AC (I)	[25] Matuszewski unpubl.
81.	***Hydrotaea aenescens***	(Wiedemann, 1830)	A/O, NA, NT, PA	HC (A); AC (A, I)	[3, 7, 8, 12, 14, 24, 27, 29, 30, 34, 37, 38, 41, 43–53]
82.	*Hydrotaea albuquerquei*	Lopes, 1985	NT	AC (A)	[8, 12, 14, 31, 35, 49, 52]
83.	*Hydrotaea anxia*	(Fallén, 1825)	NA, PA	AC (A)	[28]
84.	***Hydrotaea armipes***	(Fallén, 1825)	NA, OR, PA	AC (A, I)	[6, 24, 27, 28, 32, 37, 38, 41, 48, 54, 55]
85.	*Hydrotaea basdeni*	Collin, 1939	NA, PA	AC (A)	Jarmusz unpubl.
86.	*Hydrotaea borussica*	Stein, 1899	PA	AC (A)	Matuszewski unpubl.
87.	***Hydrotaea capensis***	(Wiedemann, 1818)	AF, OR, PA	HC (A, I); AC (A, I)	[6, 8, 14, 27, 37, 38, 47, 56–63]
88.	*Hydrotaea chalcogaster*	(Wiedemann, 1824)	AF, A/O, NA, NT, OR, PA	HC (A, I) AC (A, I)	[2, 3, 5–9, 11–14, 16, 34, 64–68]

89.	*Hydrotaea cyrtoneurina*	(Zetterstedt, 1845)	PA	AC (A)	[24, 28, 37, 38, 48, 69]
90.	***Hydrotaea dentipes***	(Fabricius, 1805)	NA, NT, OR, PA	HC (I); AC (A, I)	[24, 27, 28, 38–40, 47, 54, 62, 69–78]
91.	*Hydrotaea diabolus*	(Harris, [1780])	NA, PA	AC (A)	Matuszewski unpubl.
92.	*Hydrotaea floccosa*	Macquart, 1835	NA, OR, PA	AC (A)	[24]
93.	*Hydrotaea houghi*	Malloch, 1916	NA, NT	AC (A)	[32, 79]
94.	***Hydrotaea ignava***	(Harris, [1780])	NA, OR, PA	HC (I); AC (A, I)	[4, 24, 27, 28, 32, 37, 38, 40, 41, 47, 48, 54, 55, 64, 69, 75–78, 80–89]
95.	*Hydrotaea irritans*	(Fallén, 1823)	PA	AC (A)	[54] Matuszewski unpubl.
96.	*Hydrotaea meteorica*	(Linnaeus, 1758)	NA, PA	AC (A)	[24, 37]
97.	*Hydrotaea nicholsoni*	Curran, 1939	NT	AC (A)	[8, 31, 35]
98.	*Hydrotaea obscurifrons*	(Sabrosky, 1949)	OR, PA	CR; HC (I)	[90]
99.	*Hydrotaea palaestrica*	(Meigen, 1826)	NA, PA	AC (A)	[39, 41, 48, 54, 79]
100.	*Hydrotaea pellucens*	Portschinsky, 1879	PA	AC (A)	Matuszewski unpubl.
101.	***Hydrotaea pilipes***	Stein, 1903	PA	AC (A, I)	[24, 27, 28, 37, 54, 69, 78]
102.	***Hydrotaea similis***	Meade, 1887	PA	AC (A, I)	[24, 27, 28, 36, 37, 54, 69, 77, 78, 85]
103.	*Hydrotaea solitaria*	(Albuquerque, 1958)	NT	AC (A)	[14, 91]
104.	*Hydrotaea spinigera*	(Stein, 1910)	A/O, OR, PA	HC (I); AC (A, I)	[2, 9, 16, 18, 23, 66, 92–96]
105.	*Hydrotaea villosa*	Stein, 1904	NT	HC (A)	[49]
106.	***Hydrotaea* sp.**	Robineau-Desvoidy, 1830	AF, A/O, NA, NT, OR, PA	HC (I); AC (A, I)	[8, 13–15, 25–27, 73, 81, 87, 89, 97–105]
107.	*Limnophora exigua*	(Wiedemann, 1830)	AF, A/O, OR, PA	HC (A)	[2]
108.	*Limnophora deleta*	(van der Wulp, 1896)	NT	HC (A)	[12, 14]
109.	*Limnophora nigripes*	(Robineau-Desvoidy, 1830)	NA, PA	AC (A)	[24]
110.	*Limnophora uniseta*	Stein, 1916	NA, PA	AC (A)	[28]
111.	*Limnophora* sp.	Robineau-Desvoidy, 1830	AF, A/O, NA, NT, OR, PA	AC (A)	[25]
112.	*Lispe tentaculata*	(De Geer, 1776)	NA, NT, OP, PA	AC (A)	[24]
113.	*Lophosceles cinereiventris*	(Zetterstedt, 1845)	NA, PA	HC (A)	Jarmusz unpubl.
114.	*Mesembrina meridiana*	(Linnaeus, 1758)	PA	HC (A)	[33]
115.	*Morellia basalis*	(Walker, 1853)	NA, NT	AC (A)	[8]
116.	*Morellia couriae*	Pamplona, 1986	NT	HC (A)	[12]
117.	*Morellia dendropanacis*	Pamplona et Couri, 1995	NT	AC (A)	[8]
118.	*Morellia hortorum*	(Fallén, 1817)	PA	AC (A)	Feddern unpubl.

119. *Morellia humeralis*	Stein, 1918	NT	AC (A)	[8, 12, 14, 67]
120. *Morellia obscuripes*	(Bigot, 1887)	NT	AC (A)	[7, 12]
121. *Morellia micans*	(Macquart, 1855)	NA	AC (A)	[32]
122. *Morellia* sp.	Robineau-Desvoidy, 1830	AF, A/O, NA, NT, OR, PA	AC (A, I)	[8, 9, 27, 44, 81, 99] Matuszewski unpubl.
123. *Morellia violacea*	(Robineau-Desvoidy, 1830)	NT	AC (A)	[14]
124. ***Musca autumnalis***	De Geer, 1776	NA, NT, OR, PA	HC (I); AC (A; I)	[27, 47, 73, 106, 107] Matuszewski unpubl.
125. ***Musca domestica***	Linnaeus, 1758	AF, A/O, NA, NT, OR, PA	HC (A, I); AC (A, I)	[4–8, 11–14, 16, 18, 21, 22, 27, 29, 30, 32, 34, 38, 41, 43–50, 52, 55, 58, 64–68, 83, 84, 88, 89, 93, 96, 104, 107–115]
126. *Musca pattoni*	Austen, 1910	OR, PA	AC (A)	[6]
127. *Musca sorbens*	Wiedemann, 1830	AF, OR, PA	HC (A); AC (A)	[2, 4, 6, 15, 16, 18, 64, 116]
128. ***Musca* sp.**	Linnaeus, 1758	AF, A/O, NA, NT, OR, PA	HC (I); AC (A)	[86, 105, 117]
129. *Musca ventrosa*	Wiedemann, 1830	AF, A/O, OR	AC (A)	[6, 15, 66]
130. *Musca vetustissima*	Walker, 1849	A/O	HC (A); AC (A)	[21, 22, 68]
131. ***Muscina levida***	(Harris, [1780])	NA, NT, PA, Hawaii	HC (I); AC (A, I)	[24, 27, 28, 32, 36–39, 41–44, 47, 48, 54, 55, 70–72, 74, 77, 115, 118, 119]
132. *Muscina pascuorum*	(Meigen, 1826)	NA, OR, PA	AC (A)	[24, 27, 37, 115]
133. ***Muscina prolapsa***	(Harris, [1780])	NA, PA	AC (A, I)	[4, 24, 27, 28, 36–38, 42, 43, 48, 54, 55, 69, 75, 80, 82, 120, 121]
134. ***Muscina* sp.**	Robineau-Desvoidy, 1830	AF, A/O, NA, NT, OR, PA	HC (I); AC (A, I)	[101–103, 105, 106, 114, 122, 123]
135. ***Muscina stabulans***	(Fallén, 1817)	AF, A/O, NA, NT, OR, PA	HC (A, I); AC (A, I)	[4, 14, 24, 25, 28, 32, 34, 36–38, 41, 43, 44, 46, 52–55, 82, 102, 114, 115, 124–127]
136. *Mydaea ancilla*	(Meigen, 1826)	PA	AC (A)	[24, 36, 54] Matuszewski unpubl.
137. *Mydaea corni*	(Scopoli, 1763)	PA	AC (A)	[24] Jarmusz unpubl.; Matuszewski unpubl.
138. *Mydaea humeralis*	Robineau-Desvoidy, 1830	PA	AC (A)	[33]
139. *Mydaea nebulosa*	(Stein, 1893)	PA	AC (A)	[37, 39]
140. *Mydaea nubivena*	Snyder, 1941	NT	AC (A)	[14]
141. *Mydaea plaumanni*	Snyder, 1941	NT	AC (A)	[14, 34]
142. *Mydaea* sp.	Robineau-Desvoidy, 1830	NA, NA, PA	AC (A)	[27]
143. *Mydaea lateritia*	(Rondani, 1866)	PA	AC (A)	[128] Jarmusz unpubl.
144. *Mydaea urbana*	(Meigen, 1826)	NA, PA	AC (A)	[24, 33, 36, 39]

145. *Myospila fluminensis*	Couri & Lopes, 1988	NT	AC (A)	[14]
146. *Myospila meditabunda*	(Fabricius, 1781)	NA, NT, OR, PA	AC (A)	[24, 39, 48] Matuszewski unpubl.
147. *Myospila obscura*	(Shannon et Del Ponte, 1926)	NT	AC (A)	[34]
148. *Myospila pallidicornis*	Bigot, 1887	NT	AC (A)	[12]
149. *Myospila sp.*	Rondani, 1856	AF, A/O, NA, NT, OR, PA	AC (A)	[7, 27]
150. *Neohydrotaea sp.*	Malloch, 1924	NA, PA	AC (A)	[103]
151. *Neohydrotaea lundbecki*	(Michelsen, 1978)	PA	AC (A)	[129]
152. *Neomuscina currani*	Snyder, 1949	NT	AC (A)	[14]
153. *Neomuscina inflexa*	(Stein, 1918)	NT	AC (A)	[14]
154. *Neomuscina* sp.	Townsend, 1919	NT	AC (A)	[12, 14, 31, 35]
155. *Neomuscina tinctinervis*	(Stein, 1918)	NT	AC (A)	[14]
156. *Neurotrixa felsina*	(Walker, 1849)	NT	AC (A)	[34]
157. *Parapyrellia maculipennis*	(Macquart, 1846)	NT	AC (A; I)	[14, 30, 67]
158. *Phaonia angelicae*	(Scopoli, 1763)	PA	AC (A)	[36] Matuszewski unpubl.
159. *Phaonia errans*	(Meigen, 1826)	NA, PA	AC (A)	[28, 37]
160. *Phaonia erronea*	(Schnabl, 1887)	PA	AC (A)	[28, 36]
161. *Phaonia falleni*	Michelsen, 1977	PA	AC (A)	Matuszewski unpubl.
162. *Phaonia fuscata*	(Fallén, 1825):	OR, PA	AC (A)	Fremdt unpubl.
163. *Phaonia incana*	(Wiedemann, 1817)	OR, PA	AC (A)	[33]
164. *Phaonia mediterranea*	Hennig, 1963	PA	AC (A)	[38]
165. *Phaonia mystica*	(Meigen, 1826):	PA	AC (A)	Fremdt unpubl.
166. *Phaonia pallida*	(Fabricius, 1787)	PA	AC (A)	[28, 36–38, 54] Matuszewski unpubl.
167. *Phaonia palpata*	(Stein, 1897)	OR, PA	AC (A)	Jarmusz unpubl.
168. *Phaonia rufiventris*	(Scopoli, 1763)	PA	AC (A)	Jarmusz unpubl.
169. *Phaonia rufipalpis*	(Macquart, 1835)	PA	AC (A)	[33] Fremdt unpubl.
170. *Phaonia subventa*	(Harris, [1780])	PA	AC (A)	[24, 28, 36–38, 54]
171. *Phaonia trimaculata*	(Bouché, 1834)	PA	AC (A)	Farinha unpubl.
172. *Phaonia tuguriorum*	(Scopoli, 1763)	PA	AC (A)	[36]
173. *Phaonia valida*	(Harris, [1780])	PA	AC (A)	[28, 37, 55]
174. ***Phaonia* sp.**	Robineau-Desvoidy, 1830	AF, A/O, NA, NT, OR, PA	AC (A, I)	[8, 14, 27, 66, 130]
175. *Philornis sp.*	Meinert, 1890	NT	AC (A)	[10]

176.	*Polietes lardarius*	(Fabricius, 1781)	AF, PA	AC (A)	[37–39]
177.	*Polietina sp.*	Schnabl et Dziedzicki, 1911	NA, NT	AC (A)	[8]
178.	*Potamia littoralis*	Robineau-Desvoidy, 1830	NA, OR, PA	AC (A)	[24, 28, 54] Fremdt unpubl.
179.	*Potamia* sp.	Robineau-Desvoidy, 1830	NA, NT, OR, PA	HC (A)	[40, 105]
180.	*Pseudoptilolepis nigripoda*	Snyder, 1949	NT	AC (A)	[7]
181.	*Pseudoptilolepis* sp.	Snyder, 1949	NT	AC (A)	[14]
182.	*Psilochaeta chalybea*	(Wiedemann, 1830)	NT	AC (A)	[34]
183.	*Psilochaeta pampiana*	(Shannon et Del Ponte, 1926)	NT	AC (A)	[31, 34, 52]
184.	*Pyrellia rapax*	(Harris, [1780])	PA	AC (A)	Matuszewski unpubl.
185.	*Pyrellia vivida*	Robineau-Desvoidy, 1830	OR, PA	AC (A)	[33]
186.	*Pyrellia* sp.	Robineau-Desvoidy, 1830	AF, A/O, NA, OR, PA,	HC (A)	[105] Matuszewski unpubl.
187.	*Sarcopromusca pruna*	Shannon et Del Ponte, 1926	NT	AC (A, I)	[7, 14]
188.	*Schoenomyza litorella*	(Fallén, 1823)	AF, NA, OR, PA	AC (A)	Matuszewski unpubl
189.	*Spilogona* sp.	Schnabl, 1911	AF, A/O, NA, NT, OR, PA	AC (A)	Jarmusz unpubl.
190.	***Stomoxys calcitrans***	(Linnaeus, 1758)	AF, A/O, NA, NT, OR, PA	AC (A)	[7, 8, 21, 27, 29, 32, 52, 131]
191.	***Stomoxys* sp.**	Geoffroy, 1762	AF, A/O, NA, NT, OR, PA	HC (I)	[105]
192.	*Syllimnophora atrovittata*	Stein, 1904	NT	AC (A)	[25]
193.	***Synthesiomyia nudiseta***	(van der Wulp, 1883)	AF, A/O, NT, OR, PA	HC (A, I); AC (A; I)	[2–4, 7, 11, 12, 14, 26, 46, 47, 58, 68, 88, 92, 132–135]
194.	***Synthesiomyia* sp.**	Brauer et Bergenstamm, 1893	AF, A/O, NT, OR, PA	AC (A)	[8, 26]
195.	*Thricops diaphanus*	(Wiedemann, 1817)	NA, PA	AC (A)	[36, 54] Jarmusz unpubl.
196.	*Thricops simplex*	(Wiedemann, 1817)	PA	AC (A)	[24, 37, 54]
197.	*Thricops* sp.	Rondani, 1856	NA, PA	AC (A)	[40, 43]
198.	*Trichomorellia nigritibia*	(Snyder, 1949)	NT	HC (A)	[49]
199.	*Trichomorellia seguyi*	(Pamplona, 1983)	NT	AC (A)	[25]

HC - recorded from human cadaver

AC - recorded from animal carcass

A - recorded as adults

I - recorded in immature stage (egg, larva, pupa)

The distribution acronyms correspond to zoogeographical regions (AF, Afrotropical; A/O, Australasian/Oceanian; OR, Oriental; NA, Nearctic; NT, Neotropical; PA, Palaearctic).

References

1. Ekanem MS (2008) Immature stages and developmental biology of two muscid flies (Cyclorrhapha: Diptera) which breed in carrion in Southern Nigeria. Zool 6:1–6.
2. Bohart GE, Gressitt JL (1951) Filth-inhabiting flies of Guam. Bernice Pauahi Bish Museum Bull 1–151.
3. Tullis K, Goff ML (1987) Arthropod succession in exposed carrion in a tropical rainforest on O'ahu Island, Hawai'i. J Med Entomol 24:332–9.
4. Tantawi TI, El-Kady EM, Greenberg B, El-Ghaffar HA (1996) Arthropod succession on exposed rabbit carrion in Alexandria, Egypt. J Med Entomol 33:566–580.
5. Avila FW, Goff ML (1998) Arthropod succession patterns onto burnt carrion in two contrasting habitats in the Hawaiian Islands. J Forensic Sci 43:581–6.
6. Bharti M, Singh D (2003) Insect faunal succession on decaying rabbit carcasses in Punjab, India. J Forensic Sci 48:1133–43.
7. Rosa TA, Babata MLY, Souza CM de, et al (2011) Arthropods associated with pig carrion in two vegetation profiles of Cerrado in the State of Minas Gerais, Brazil. Rev Bras Entomol 55:424–434.
8. Ramírez-Mora MA, Buenaventura E, Gómez-P LM, Amat E (2012) Updated checklist and new records of Calyptratae carrion flies (Diptera, Schizophora) from Valle de Aburrá and other localities in Colombia. Entomotropica 27:27–35.
9. Azwandi A, Nina Keterina H, Owen LC, et al (2013) Adult carrion arthropod community in a tropical rainforest of Malaysia: analysis on three common forensic entomology animal models. Trop Biomed 30:481–94.
10. Alves ACF, dos Santos WE, Creão-Duarte AJ (2014) Diptera (Insecta) de importância forense da região Neotropical. Entomotropica 29:77–94.
11. Vasconcelos SD, Barbosa TM, Oliveira TPB (2015) Diversity of forensically-important dipteran species in different environments in northeastern Brazil, with notes on the attractiveness of animal baits. Florida Entomol 98:770–775.
12. Barbosa RR, Mello-Patiu CA de, Mello RP de, Queiroz MM de C (2009) New records of calyptrate dipterans (Fanniidae, Muscidae and Sarcophagidae) associated with the decomposition of domestic pigs in Brazil. Memórias do Inst Oswaldo Cruz 104:923–6.
13. Moretti T de C, Solis DR, Linhares AX, et al (2009) 12 - month survey of necrophagous insects in Southeastern Brazil. In: Proc. Seventh Meet. Eur. Assoc. Forensic Entomol. Uppsala, Sweden, pp 51–52
14. Cavallari ML, Baltazar FN, Nihei SS, et al (2015) Dipterofauna associated with *Sus scrofa* Linné , 1758 , Carcasses in urban and coastal regions of São Paulo State , Brazil. Psyche (Stuttg).
15. Sukontason K, Sukontason KL, Piangjai S, et al (2003) Survey of forensically-relevant fly species in Ciang Mai, Thailand. J Vector Ecol 28:135–138.
16. Silahuddin SA, Latif B, Kurahashi H, et al (2015) The importance of habitat in the ecology of decomposition on rabbit carcasses in Malaysia: implications in forensic entomology. J Med Entomol 52:9–23.
17. Amendt J, Richards CS, Campobasso CP, et al (2011) Forensic entomology: Applications and limitations. Forensic Sci Med Pathol 7:379–392.
18. Sukchit M, Deowanish S, Butcher BA (2015) Decomposition stages and carrion insect succession on dressed hanging pig carcasses in Nan Province, Northern Thailand. Trop Nat Hist 15:137–153.
19. Archer MS, Elgar MA (2003) Effects of decomposition on carcass attendance in a guild of carrion-breeding flies. Med Vet Entomol 17:263–271.
20. Eberhardt TL, Elliot DA (2008) A preliminary investigation of insect colonisation and succession on remains in New Zealand. Forensic Sci Int 176:217–23.
21. Voss SC, Forbes SL, Dadour IR (2008) Decomposition and insect succession on cadavers inside a vehicle environment. Forensic Sci Med Pathol 4:22–32.

22. Voss SC, Cook DF, Dadour IR (2011) Decomposition and insect succession of clothed and unclothed carcasses in Western Australia. Forensic Sci Int 211:67–75.
23. O`Flynn MA (1983) The succession and rate of development of blowflies in carrion in southern Queensland and the application of these data to forensic entomology. Aust J Entomol 22:137–148.
24. Klimešová V, Slobodová M, Šuláková H, Miroslav B (2014) Use of the family Muscidae (Diptera) in forensic practice. 6th Work. Biodivers.
25. Martinez E, Duque P, Wolff M (2007) Succession pattern of carrion-feeding insects in Paramo, Colombia. Forensic Sci Int 166:182–189.
26. Segura NA, Usaquen W, Sanchez MC, et al (2009) Succession pattern of cadaverous entomofauna in a semi-rural area of Bogota, Colombia. Forensic Sci Int 187:66–72.
27. Diaz Martin B (2015) Entomofauna associated with domestic pig (*Sus scrofa*) decomposition in an Atlantic Environment (Aiako Harria, Basque Country, Spain). Universidad del Pais Vasco
28. Fiedler A, Halbach M, Sinclair B, Benecke M (2008) What is the Edge of a Forest? A Diversity Analysis of Adult Diptera Found on Decomposing Piglets inside and on the Edge of a Western German Woodland inspired by a Courtroom Question. Entomol heute 20:173–191.
29. Grisales D, Magnolia R, Villegas S (2010) Insects associated with exposed decomposing bodies in the Colombian Andean Coffee Region. Rev Bras Entomol 54:637–644.
30. Faria LS, Paseto ML, Franco FT, et al (2013) Insects breeding in pig carrion in two environments of a rural area of the state of minas gerais, Brazil. Neotrop Entomol 42:216–22.
31. Moura MO, de Carvalho CJ, Monteiro-Filho EL (1997) A preliminary analysis of insects of medico-legal importance in Curitiba, State of Paraná. Memórias do Inst Oswaldo Cruz 92:269–74.
32. Payne JA, King EW (1972) Insect succession and decompostition of pig carcasses in water. J Georg Entomol Soc 7:153–162.
33. Skidmore P (1985) The biology of the Muscidae of the World. Ser Entomol 29:1–550.
34. Mulieri PR, Patitucci LD, Olea MS (2015) Sex-biased patterns of saprophagous Calyptratae (Diptera) collected with different baits of animal origin. J Med Entomol 52:386–93.
35. Moura MO, Carvalho CJB de, Monteiro-Filho EL (1998) Carrion attendant arthropods in southern Brazil. Cienc Cult 50:377–381.
36. Hwang C, Turner BD (2005) Spatial and temporal variability of necrophagous Diptera from urban to rural areas. Med Vet Entomol 19:379–391.
37. Matuszewski S, Bajerlein D, Konwerski S, Szpila K (2008) An initial study of insect succession and carrion decomposition in various forest habitats of Central Europe. Forensic Sci Int 180:61–69.
38. Baz A, Cifrián B, Martín-Vega D (2014) Patterns of diversity and abundance of carrion insect assemblages in the natural park “Hoces del Río Riaza” (Central Spain). J Insect Sci 14:1–10.
39. Hanski I (1976) Breeding experiments with carrion flies (Diptera) in natural conditions. Ann Entomol Fenn 42:113–121.
40. Watson EJ, Carlton CE (2005) Insect succession and decomposition of wildlife carcasses during fall and winter in Louisiana. J Med Entomol 42:193–203.
41. Miralbes MC (2002) Estudio de la entomofauna asociada a cadáveres en el Alto Aragón (España). Monogr SEA 6:94.
42. Blackith RE, Blackith RM (1990) Insect infestations of small corpses. J Nat Hist 24:699–709.
43. Arnaldos I, Romera E, García MD, Luna A (2001) An initial study on the succession of sarcosaprophagous Diptera (Insecta) on carrion in the southeastern Iberian peninsula. Int J Legal Med 114:156–62.

44. Centeno N, Maldonado M, Oliva A (2002) Seasonal patterns of arthropods occurring on sheltered and unsheltered pig carcasses in Buenos Aires Province (Argentina). Forensic Sci Int 126:63–70.
45. Bucheli SR, Bytheway JA, Pustilnik SM, Florence J (2009) Insect successional pattern of a corpse in cooler months of subtropical southeastern Texas. J Forensic Sci 54:452–5.
46. Horenstein MB, Linhares AX, De Ferradas BR, García D (2010) Decomposition and dipteran succession in pig carrion in central Argentina: ecological aspects and their importance in forensic science. Med Vet Entomol 24:16–25.
47. Velásquez Y, Magaña C, Martínez-Sánchez A, Rojo S (2010) Diptera of forensic importance in the Iberian Peninsula: larval identification key. Med Vet Entomol 24:293–308.
48. Anton E, Niederegger S, Beutel RG (2011) Beetles and flies collected on pig carrion in an experimental setting in Thuringia and their forensic implications. Med Vet Entomol 25:353–64.
49. Barrios M, Wolff M (2011) Initial study of arthropods succession and pig carrion decomposition in two freshwater ecosystems in the Colombian Andes. Forensic Sci Int 212:164–72.
50. Ramos-Pastrana Y, Velasquez-Valencia A, Wolff M (2014) Preliminary study of insects associated to indoor body decay in Colombia. Rev Bras Entomol 58:326–332.
51. Rysavy NM, Goff ML (2015) Preliminary observations of arthropods associated with buried carrion on Oahu. J Forensic Sci 60:462–7.
52. Silva ÂZ da, Hoffmeister CH, Anjos VA dos, et al (2014) Necrophagous diptera associated with wild animal carcasses in southern Brazil. Rev Bras Entomol 58:337–342.
53. Mariani R, García-Mancuso R, Varela GL, Inda AM (2014) Entomofauna of a buried body: study of the exhumation of a human cadaver in Buenos Aires, Argentina. Forensic Sci Int 237:19–26.
54. Kentner E, Streit B (1990) Temporal and habitat preference of congeneric insect species found at rat carrion. Pedobiologia (Jena) 34:347–359.
55. Baz A, Botías C, Martín-Vega D, et al (2015) Preliminary data on carrion insects in urban (indoor and outdoor) and periurban environments in central Spain. Forensic Sci Int 248:41–7.
56. Braack LEO (1987) Community dynamics of carrion-attendant arthropods in tropical african woodland. Oecologia 72:402–409.
57. Bourel B, Tournel G, Hédouin V, Gosset D (2004) Entomofauna of buried bodies in northern France. Int J Legal Med 118:215–20.
58. Pérez SP, Duque P, Wolff M (2005) Successional behavior and occurrence matrix of carrion-associated arthropods in the urban area of Medellín, Colombia. J Forensic Sci 50:448–54.
59. Suláková H, Markvartová J, Beran M (2013) Insect and a dead man in his flat. Case report. Soud Lek 58:2–5.
60. Turchetto M, Vanin S (2004) Forensic evaluations on a crime case with monospecific necrophagous fly population infected by two parisitoid species. Aggrawal's J Forensic Med Toxicol 5:12–18.
61. García-Rojo AM, Honorato L, González M, Téllez A (2009) Determinación del intervalo postmortem mediante el estudio de la sucesión de insectos en dos cadáveres hallados en el interior de una finca rústica en Madrid. Cuad Med Forense 56:137–145.
62. Vanin S, Gherardi M (2009) A review of 200 forensic cases involving insects from Northern Italy. In: Proc. Seventh Meet. Eur. Assoc. Forensic Entomol. Uppsala, Sweden, pp 33–34

63. Morrow JJ, Baldwin DA, Higley L, et al (2015) Curatorial implications of *Ophyra capensis* (Order Diptera, Family Muscidae) puparia recovered from the body of the Blessed Antonio Patrizi, Monticiano, Italy (Middle Ages). J Forensic Leg Med 36:81–83.
64. Shalaby OA, deCarvalho LM, Goff ML (2000) Comparison of patterns of decomposition in a hanging carcass and a carcass in contact with soil in a xerophytic habitat on the Island of Oahu, Hawaii. J Forensic Sci 45:1267–73.
65. Carvalho LML, Thyssen PJ, Goff ML, Linhares AX (2004) Observations on the succession patterns of necrophagous insects on a pig carcass in an urban area of Southeastern Brazil. Anil Aggrawal's Internet J Forensic Med Toxicol 5:33–39.
66. Wang J, Li Z, Chen Y, et al (2008) The succession and development of insects on pig carcasses and their significances in estimating PMI in south China. Forensic Sci Int 179:11–8.
67. Vasconcelos SD, Cruz TM, Salgado RL, Thyssen PJ (2013) Dipterans associated with a decomposing animal carcass in a rainforest fragment in Brazil: notes on the early arrival and colonization by necrophagous species. J Insect Sci 13:145.
68. Farrell JF, Whittington AE, Zalucki MP (2015) A review of necrophagous insects colonising human and animal cadavers in south-east Queensland, Australia. Forensic Sci Int 257:149–54.
69. Mądra A, Frątczak K, Grzywacz A, Matuszewski S (2015) Long-term study of pig carrion entomofauna. Forensic Sci Int 252:1–10.
70. Nuorteva P (1977) Sarcosaprophagous insects as forensic indicators. In: Tedeschi CG, Eckert WG, Tedeschi LG (eds) A study trauma Environ. hazards. Saunders, Philadelphia, pp 1072–1095
71. Kunsela S, Hanski I (1982) The structure of carrion fly communities: the size and the type of carrion. Holarct Ecol 5:337–348.
72. Hanski I (1987) Carrion fly community dynamics: patchiness, seasonality and coexistance. Ecol Entomol 12:257–266.
73. Anderson GS, VanLaerhoven S (1996) Initial studies on insect succession on carrion in Southwestern British Columbia. J Forensic Sci 41:617–625.
74. Kouki J, Hanski I (1995) Population aggregation facilitates coexistence of many competing carrion fly species. Oikos 72:223–227.
75. Bourel B, Martin-Bouyer L, Hedouin V, et al (1999) Necrophilous insect succession on rabbit carrion in sand dune habitats in northern France. J Med Entomol 36:420–5.
76. Wyss C, Cherix D (2006) Trait´e d'Entomologie Forensique. Les Insectes sur la Scène de crime. Presses Polytechniques et Universitaires Romandes, Lausana
77. Grzywacz A, Lindström A, Hall MJR (2014) *Hydrotaea similis* Meade (Diptera: Muscidae) newly reported from a human cadaver: A case report and larval morphology. Forensic Sci Int 242:e34–e43.
78. Matuszewski S, Bajerlein D, Konwerski S, Szpila K (2010) Insect succession and carrion decomposition in selected forests of Central Europe. Part 2: Composition and residency patterns of carrion fauna. Forensic Sci Int 195:42–51.
79. Jong De GD, Chadwick JW (1999) Decomposition and arthropod succession on exposed rabbit carrion during summer at high altitudes in Colorado, USA. J Med Entomol 36:833–845.
80. Lundt H (1964) Ökologische Untersuchungen über die tierische Besiedlung von Aas im Boden. Pedobiologia (Jena) 4:158–180.
81. VanLaerhoven S, Anderson GS (1999) Insect succession on buried carrion in two biogeoclimatic zones of British Columbia. J Forensic Sci 44:32–43.
82. Grassberger M, Frank C (2004) Initial study of arthropod succession on pig carrion in a central European urban habitat. J Med Entomol 41:511–23.
83. Tabor KL, Brewster CC, Fell RD (2004) Analysis of the successional patterns of insects on carrion in southwest Virginia. J Med Entomol 41:785–95.

84. Tabor KL, Fell RD, Brewster CC (2005) Insect fauna visiting carrion in Southwest Virginia. Forensic Sci Int 150:73–80. doi: 10.1016/j.forsciint.2004.06.041
85. Boehme P, Amendt J, Zehner R (2012) The use of COI barcodes for molecular identification of forensically important fly species in Germany. Parasitol Res 110:2325–2332.
86. Sert O, Kabalak M, Sabanoglu B (2012) Determination of forensically important Coleoptera and Calliphoridae (Diptera) species on decomposing dog (*Canis lupus famaliaris* L.) carcass at Ankara Province. Hacettepe J Biol Chem 40:99–103.
87. Pastula EC, Merritt RW (2013) Insect arrival pattern and succession on buried carrion in Michigan. J Med Entomol 50:432–439.
88. Richards SL, Connelly CR, Day JF, et al (2015) Arthropods associated with carrion in a salt marsh habitat in Southeastern Florida. Florida Entomol 98:613–619.
89. Anderson GS (1995) The use of insects in death investigations: an analysis of cases in British Columbia over a five year period. Can Soc Forensic Sci J 28:277–292.
90. Shin SE, Lee HJ, Park JH, et al (2015) The first survey of forensically important entomofauna collected from medicolegal autopsies in South Korea. Biomed Res Int.
91. Moretti T de C, Ribeiro OB, Thyssen PJ, Solis DR (2008) Insects on decomposing carcasses of small rodents in a secondary forest in Southeastern Brazil. Eur J Entomol 105:691–696.
92. Sukontason K, Narongchai P, Kanchai C, et al (2007) Forensic entomology cases in Thailand: a review of cases from 2000 to 2006. Parasitol Res 101:1417–23.
93. Rajagopal K, Lim LH, Dhang CC, et al (2009) Comparison of forensically important fly larvae recovered from human cadavers and monkey cadavers in Malaysia. In: Proc. seventh Meet. Eur. Assoc. Forensic Entomol. pp 52–53
94. Heo CC, Mohamad AM, John J, Baharudin O (2008) Insect succession on a decomposing piglet carcass placed in a man-made freshwater pond in Malaysia. Trop Biomed 25:23–9.
95. Heo CC, Mohamad AM, Ahmad FMS, et al (2008) Study of insect succession and rate of decomposition on a partially burned pig carcass in an oil palm plantation in Malaysia. Trop Biomed 25:202–8.
96. Chen CD, Lee HL, Nazni WA, et al (2010) First report of the house fly larvae, *Musca domestica* (Linnaeus) (Diptera: Muscidae) associated with the monkey carcass in Malaysia. Trop Biomed 27:355–9.
97. Marchiori CH, Silva CG, Caldas ER, et al (2000) Artrópodos associados com a carcaça de suíno em Itumbiara, sul de Goiás. Arq Inst Biol (Sao Paulo) 67:167–170.
98. Oliva A (2001) Insects of forensic significance in Argentina. Forensic Sci Int 120:145–54.
99. Wolff M, Uribe A, Ortiz A, Duque P (2001) A preliminary study of forensic entomology in Medellin, Colombia. Forensic Sci Int 120:53–59. doi:
100. Grassberger M, Scharrer-Liška G (2006) Archaeoentomology: examination and interpretation of entomological specimens from a 1200-year-old burial site in Austria. In: Proc. Fourth Meet. Eur. Assoc. Forensic Entomol. Bari, Italy, p 71
101. Gaudry E, Dourel L, Chauvet B, et al (2007) Effect of burial on necrophagous insect activity. In: Proc. fifth Meet. Eur. Assoc. Forensic Entomol. p 15
102. Schoenly KG, Haskell NH, Hall RD, Gbur JR (2007) Comparative performance and complementarity of four sampling methods and arthropod preference tests from human and porcine remains at the Forensic Anthropology Center in Knoxville, Tennessee. J Med Entomol 44:881–894.
103. Bygarski K, Leblanc HN (2013) Decomposition and arthropod succession in Whitehorse, Yukon territory, Canada. J Forensic Sci 58:413–418.
104. Mabika N, Masendu R, Mawera G (2014) An initial study of insect succession on decomposing rabbit carrions in Harare, Zimbabwe. Asian Pac J Trop Biomed 4:561–5.
105. Lefebvre F, Gaudry E (2009) Forensic entomology: a new hypothesis for the chronological succession pattern of necrophagous insect on human corpses. Ann la Société

Entomol Fr 45:377–392.
106. Smith KG V. (1986) A Manual of Forensic Entomology. British Museum (Natural History), London, and Cornell University Press, Ithaca, NY
107. Mohamed Aly S, Wen J, Wang X, et al (2013) Identification of forensically important arthropods on exposed remains during summer season in northeastern Egypt. Zhong Nan Da Xue Xue Bao Yi Xue Ban 38:1–6.
108. Baldridge RS, Wallace SG, Kirkpatrick R (2006) Investigation of nocturnal oviposition by necrophilous flies in central Texas. J Forensic Sci 51:125–6.
109. Haskell NH, Tomberlin J (2006) Disparity between eyewitness accounts of a murder in Texas and the recovery and analysis of entomological evidence. In: Proc. Fourth Meet. Eur. Assoc. Forensic Entomol. Bari, Italy, p 48
110. Heo CC, Mohamad AM, Jeffery J, et al (2008) On the occurrence of *Musca domestica* L oviposition activity on pig carcass in peninsular Malaysia. Trop Biomed 25:252–3.
111. Barreto M, Burbano ME, Barreto P (2002) Flies (Calliphoridae, Muscidae) and Beetles (Silphidae) from Human Cadavers in Cali, Colombia. Mem Inst Oswaldo Cruz, Rio Janeiro 97:137–138.
112. Perveen F, Khan A (2013) Ecological succession pattern of the insect species associated with the carcass of the free-ranging urban dog, *Canis domesticus* (L.): a tool for forensic entomology. Mintage J Pharm Med Sci 2:31–36.
113. Gurafi LMA, Mohamed AR (2012) Decomposition and arthropod succession on two monkeys (*Cercopithecus aethiopicus*). Sudan J Sci 5:1–7.
114. Arnaldos MI, Garcia MD, Romera E, et al (2005) Estimation of postmortem interval in real cases based on experimentally obtained entomological evidence. Forensic Sci Int 149:57–65.
115. Iancu L, Carter DO, Junkins EN, Purcarea C (2015) Using bacterial and necrophagus insect dynamics for post-mortem interval estimtion during cold seaon - Novel case study in Romania. Forensic Sci Int 254:106–117.
116. Al-Mesbah H, Al-Osaimi Z, El-Azazy OME (2011) Forensic entomology in Kuwait: the first case report. Forensic Sci Int 206:e25–6.
117. Goff ML, Odom CB (1987) Forensic entomology in the Hawaiian Islands. Three case studies. Am J Forensic Med Pathol 8:45–50.
118. Greenberg B, Kunich JC (2002) Entomology and the law: Flies as forensic indicators. Cambridge University Press, Cambridge
119. Iancu (Paul) L, Pârvu C (2013) Necrophagous entomofauna (Diptera, Coleoptera) on *Meles meles* (L.) (Mammalia: Mustelidae) carcasses within different conditions of exposure in Bucharest (Romania). Trav du Muséum Natl d’Histoire Nat Grigore Antipa" 56:45–63.
120. Szpila K, Voss JG, Pape T (2010) A new dipteran forensic indicator in buried bodies. Med Vet Entomol 24:278–283.
121. Bernhardt V, Fremdt H, Huibregts H, et al (2016) *Muscina prolapsa* (Harris 1780). Rechtsmedizin 26:97–102.
122. Petrašiūnas A (2004) Insect colonization of rat carrion under rural and urban conditions in Lithuania. Forensic Sci Int 169S:S27–S28.
123. Gaudry E (2010) The insects colonization of buried remains. In: Amendt J, Goff ML, Campobasso CP, Grassberger M (eds) Curr. concepts forensic Entomol. Springer, Dordrecht, pp 273–311
124. Benecke M (1998) Six forensic entomology cases: description and commentary. J Forensic Sci 43:797–805.
125. Benecke M, Lessig R (2001) Child neglect and forensic entomology. Forensic Sci Int 120:155–159.
126. Turchetto M, Lafisca S, Costantini G (2001) Postmortem interval (PMI) determined by study sarcophagous biocenoses: three cases from the province of Venice (Italy). Forensic Sci Int 120:28–31.

127. Schroeder H, Klotzbach H, Püschel K (2003) Insects' colonization of human corpses in warm and cold season. Leg Med 5:S372–S374.

128. Grzywacz A, Pape T (2010) Egg morphology of *Mydaea lateritia* (Rondani, 1866) (Diptera: Muscidae). Entomol Fenn 21:187–192.

129. Grzywacz A, Amendt J, Fremdt H (2016) Seek, and ye shall find – the example of *Neohydrotaea lundbecki* (Michelsen) (Diptera: Muscidae), a rare muscid species or just ignored so far in forensic entomology? North-Western J Zool 12:196–198.

130. Turner B, Wiltshire P (1999) Experimental validation of forensic evidence: a study of the decomposition of buried pigs in a heavy clay soil. Forensic Sci Int 101:113–22.

131. Prado e Castro C, Serrano A, Martins Da Silva P, García MD (2012) Carrion flies of forensic interest: a study of seasonal community composition and succession in Lisbon, Portugal. Med Vet Entomol 26:417–31.

132. Lord WD, Rodriguez WC (1989) Forensic entomology: the use of insects in the investigation of homicide and untimely death. Prosec 22:41–48.

133. Goff ML (2000) A fly for the prosecution: how insect evidence helps solve crimes. Harvard University Press, Cambridge

134. Lee HL, Krishnasamy M, Abdullah AG, Jeffery J (2004) Review of forensically important entomological specimens in the period of 1972 - 2002. Trop Biomed 21:69–75.

135. Calderón-Arguedas O, Troyo A, Solano ME (2005) Cuantificación de formas larvales de *Synthesiomyia nudiseta* (Diptera: Muscidae) como un criterio en el análisis del intervalo post mortem. Parasitol Latinoam 60:138–143.
