# Supplementary material for: Muscidae (Diptera) of forensic importance—an identification key to third instar larvae of the western Palaearctic region and a catalogue of the muscid carrion community
Source: Int J Legal Med. 2016 Dec 7;131(3):855–66. doi: 10.1007/s00414-016-1495-0 (PMC5388714; doi:10.1007/s00414-016-1495-0)
Supplement: Supplementary file 2 — (PDF 31.8 kb) [file 414_2016_1495_MOESM2_ESM.pdf]

Discrepancies in Muscidae third instar larva taxonomy in the literature and the present study

Hydrotaea sp. in Queiroz & Carvalho [1]

Queiroz & Carvalho [1] studied Diptera larvae inhabiting domestic dumps in Brazil. These authors also examined species and genera that are the subject of the present research. Nevertheless, specimens identified by the authors as *Hydrotaea* sp. in fact do not represent any of the Muscidae genera. This is beyond doubt because of the presence of parastomal bars and a window in the ventral cornu (Queiroz & Carvalho [1]: "FIG. 31"), a feature absent in Muscidae.

Musca sp. in Turner [2]

In his outline of forensic entomology, Turner [2] enclosed figures which supposedly present the entire body, cephaloskeleton and posterior spiracles of *Musca* sp., his figures "1, 3 and 4". Only the latter figure represents an unquestionable *Musca* sp. The illustration (Turner 1987, "Fig. 1") presenting the entire larva is devoid of a pseudocephalon. It resembles a blow fly larva rather than a housefly in the shape of the anal division and papillae surrounding the spiracular field. Turner's ([2]: "Fig. 4") drawing of the cephaloskeleton, presented in a slightly tilted position, includes only mouthhooks, dental sclerites, an intermediate sclerite and basal sclerites with a marked optic depression. The left mouthhook is of the same length as the right one and not asymmetric as is typical for *Musca* sp. The dental sclerites are fused ventrally in the form of a ventral arc, a feature not compatible with the present state of knowledge of *Musca* larvae.

Morphological diagnosis of Muscidae larvae in Gennard [3, 4]

According to Gennard [3] "the larvae of the Muscidae are recognizable by the wiggly S-shaped slits on the posterior spiracles". Gennard [3] listed only two muscids among the forensically important species of Muscidae, *Musca autumnalis* and *M. domestica*, and erroneously implied that the third instar larva of all Muscidae possesses respiratory slits of this shape. In the second edition of the textbook, Gennard [4] added *H. dentipes* as a forensically important species and broadened the diagnosis of the family into: respiratory slits "range from straight through sinuate ("s-shaped") to bowed". The "S-shape" of the respiratory slits in the posterior spiracles of Muscidae is not present in *Musca* [5]. Among species of forensic importance this shape occurs in *Stomoxys calcitrans* and *Synthesiomyia nudiseta*. The shape depicted in Gennard's [3] "Figure 2.23" and subsequently in Gennard [4] is in fact tortuous (herein Fig. 3E) not S-shaped (herein Fig. 3H) and these two states, S-shaped and tortuous, are not identical [5, 6]. Gennard [4] followed the literature concerning the third instar larvae of *H. dentipes* and stated that they "are often distinguished on the basis of the cephalopharyngeal skeleton". Since a closely related species, *H. similis*, has also been recognized as forensically important [7] and possesses a cephaloskeleton similar to the former species, this diagnosis is insufficient. The only reliable character currently known to distinguish these two species is spines above the anal papillae, absent in *H. dentipes* and present in *H. similis* [7].

Morphological diagnosis of Muscidae larvae in Byrd & Castner [8]

Byrd & Castner [8] erroneously diagnosed third instars of Muscidae as equipped with only S-shaped or sinuous slits.

Morphological diagnosis of Muscidae larvae in Thyssen [9]

Thyssen [9] in her key for the identification of immature stages of forensically important insects erroneously diagnosed all third instars of Muscidae as having only one mouthhook and sinuous respiratory slits in the posterior spiracles. Mouthhooks in Muscidae are always paired but may be symmetric or asymmetric. In the latter case the left hook is always

more or less reduced apically and asymmetric mouthhooks closely appose each other in their apical parts, whereas basal parts are joined dorsally through an apically narrow but basally broadened, unpaired sclerite (Figs 2B, C). Symmetric mouthhooks are clearly separated throughout their entire length and the unpaired sclerite is distinctly reduced into a spicule and lies freely (Fig. 2D). For this reason the former of the two characters provided by Thyssen [9] is fully misleading, whereas the latter distinguishes only larvae of the genus *Musca*.

Taxonomic identity of *Hydrotaea armipes* and *Hydrotaea floccosa* in Zimin [10], Lobanov [11], Dušek [12] and Kaczorowska & Draber-Mońko [13].

Pont [14] revealed that the species described under the name *H. floccosa* (Macquart, 1835) was erroneously recognized by authors (not Fallén) as *H. armipes* (Fallén, 1825). At the same time the true *H. armipes* was misidentified as *H. occulta* (Meigen, 1826). Therefore, any record published prior to Pont's [14] study should be treated with caution. Zimin's [10] and Lobanov's [11] descriptions of larval morphology of the species termed *H. armipes* in fact refer to *H. floccosa*. Dušek [12], who translated the key of Zimin [10], misidentified these two species as well. Kaczorowska & Draber-Mońko [13] probably missed the revision of Pont [14] and for *H. armipes* provided Zimin's [10] illustrations, which in fact present larva of *H. floccosa.*

Hydrotaea ignava in Moon [15]

Moon (2002, "Figure 14.5 E") redrew the posterior spiracle of the third instar larva from Skidmore [5] and labelled it as a "black garbage fly (*Hydrotaea ignava*)". The illustration shows parallel respiratory slits and clearly does not represent *H. ignava*. Moreover, Skidmore [5] did not provide a figure of the posterior spiracles of *H. ignava*. Moon [15] apparently misidentified the common name of *H. aenescens*, "black garbage fly", with *H. ignava*.

Mouthhook arrangement

According to Roback [16] and Ferrar [17] third instars of muscids differ from other Calyptratae by closely apposed mouthhooks. Skidmore [5] already stated that this is not the case, and this was recently confirmed in *Atherigona orientalis*, *Muscina* spp. and *S. nudiseta* [6, 18, 19]. For this reason the aforementioned proposal of Roback [16] and Ferrar [17] must be considered with caution.

Spinulation pattern

Two character states in the anterior spinose band on the first thoracic segment have been recognized herein, either uniformly broad or further ramified ventrally. Despite its utility in species differentiation of *H. aenescens* from very similar larvae of *H. capensis* and *H. ignava*, previous authors did not pay attention to this character [10, 20–22]. Although Skidmore [5] recognized both features in third instars of other larvae of Muscidae, he did not attempt to use them for taxonomic purposes, which has been done herein.

References

1. Queiroz SMP de, Carvalho CJB de (1987) Chave pictórica e descrições de larvas de 3º ínstar de Diptera (Calliphoridae, Muscidae e Fanniidae) em vazadouros de resíduos sõlidos domésticos em Curitiba, Paraná. An Soc Entomológica do Bras 16:265–288.
2. Turner B (1987) Forensic entomology: insects against crime. Sci Prog 71:133–144.
3. Gennard DE (2007) Forensic entomology: an introduction, First Edit. John Wiley & Sons Ltd., West Sussex
4. Gennard DE (2012) Forensic entomology: an introduction, Second Edi. John Wiley & Sons Ltd., West Sussex
5. Skidmore P (1985) The biology of the Muscidae of the World. Ser Entomol 29:1–550.
6. Velásquez Y, Ivorra T, Grzywacz A, et al (2013) Larval morphology, development and forensic importance of *Synthesiomyia nudiseta* (Diptera: Muscidae) in Europe: a rare species or just overlooked? Bull Entomol Res 103:98–110. doi: 10.1017/S0007485312000491
7. Grzywacz A, Lindström A, Hall MJR (2014) *Hydrotaea similis* Meade (Diptera: Muscidae) newly reported from a human cadaver: A case report and larval morphology. Forensic Sci. Int.
8. Byrd JH, Castner JL (2010) Insects of forensic importance. In: Byrd JH, Castner JL (eds) Forensic Entomol. Util. arthropods Leg. Investig. CRC Press, Boca Raton, pp 39–126
9. Thyssen PJ (2010) Keys for identification of immature insects. In: Amendt J, Goff ML, Campobasso C Pietro, Grassberger M (eds) Curr. concepts forensic Entomol. Springer, Dordrecht, pp 25–42
10. Zimin LS (1948) Opredeliteli lichinok sinantropnykh mukh Tadzhikistana. Opredeliteli po Faune CCCP 28:1–116.
11. Lobanov AM (1968) O morfologii lichinok III vozrasta sinantropnykh vidov mukh roda Hydrotaea R.-D. (Diptera, Muscidae). Zool Zhurnal 47:85–90.
12. Dusek J (1971) Key to larvae. In: Greenberg B (ed) Flies Dis. Princeton University Press, New Jersey, pp 82–88
13. Kaczorowska E, Draber-Mońko A (2009) Wprowadzenie do entomologii sądowej. Wydawnictwo Uniwersytetu Gdańskiego. Wydawnictwo Uniwersytetu Gdańskiego, Gdańsk
14. Pont AC (1984) A revision of the Fanniidae and Muscidae (Diptera) described by Fallén. Insect Syst Evol 15:277–297.
15. Moon R D (2002) Muscid Flies (Muscidae). In: Durden LA, Mullen GA (eds) Med. Vet. Entomol. Academic Press, San Diego, California, pp 279–301
16. Roback SS (1951) A classification of the muscoid Calyptrate, Diptera. Ann Entomol Soc Am 44:327–361.
17. Ferrar P (1979) The immature stages of dung-breeding muscoid flies in Australia, with notes on the species, and keys to larvae and puparia. Aust J Zool Suppl Ser 73:1–106.
18. Grzywacz A, Pape T (2014) Larval morphology of *Atherigona orientalis* (Schiner) (Diptera: Muscidae) - A species of sanitary and forensic importance. Acta Trop 137:174–184. doi: 10.1016/j.actatropica.2014.05.018
19. Grzywacz A, Hall MJR, Pape T (2015) Morphology successfully separates third instar larvae of Muscina. Med Vet Entomol 29:314–329. doi: 10.1111/mve.12117
20. Ishijima H (1967) Revision of the third stage larvae of synanthropic flies of Japan (Diptera: Anthomyiidae, Muscidae, Calliphoridae and Sarcophagidae). Japanese J Sanit Zool 18:47–100.
21. Keilin D (1917) Recherches sur les Anthomyiidaes a larves carnivores. Parasitology 9:325–450.

22. Keilin D, Tate P (1930) On certain semi-carnivorous Anthomyid larvae. Parasitology 22:168–181.
